# Demystifying estimands in cluster-randomised trials

**DOI:** 10.1177/09622802241254197

**Published:** 2024-05-23

**Authors:** Brennan C Kahan, Bryan S Blette, Michael O Harhay, Scott D Halpern, Vipul Jairath, Andrew Copas, Fan Li

**Affiliations:** 1 524254 MRC Clinical Trials Unit at UCL, Institute of Clinical Trials and Methodology, UCL, London, UK; 2 Department of Biostatistics, 12328 Vanderbilt University Medical Center, Nashville, USA; 3 Department of Biostatistics, Epidemiology, and Informatics, Perelman School of Medicine, 6572 University of Pennsylvania, Philadelphia, USA; 4 Department of Medicine, Division of Gastroenterology, Schulich School of Medicine, Western University, London, ON, Canada; 5 Department of Epidemiology and Biostatistics, Western University, London, ON, Canada; 6 Department of Biostatistics, Yale University School of Public Health, New Haven, CT, USA; 7 Center for Methods in Implementation and Prevention Science, Yale University School of Public Health, New Haven, CT, USA

**Keywords:** Estimand, cluster-randomised trial, independence estimating equations, analysis of cluster-level summaries, participant-average, cluster-average, marginal, cluster-specific

## Abstract

Estimands can help clarify the interpretation of treatment effects and ensure that estimators are aligned with the study's objectives. Cluster-randomised trials require additional attributes to be defined within the estimand compared to individually randomised trials, including whether treatment effects are *marginal* or *cluster-specific*, and whether they are *participant-* or *cluster-average*. In this paper, we provide formal definitions of estimands encompassing both these attributes using potential outcomes notation and describe differences between them. We then provide an overview of estimators for each estimand, describe their assumptions, and show consistency (i.e. asymptotically unbiased estimation) for a series of analyses based on cluster-level summaries. Then, through a re-analysis of a published cluster-randomised trial, we demonstrate that the choice of both estimand and estimator can affect interpretation. For instance, the estimated odds ratio ranged from 1.38 (*p* = 0.17) to 1.83 (*p* = 0.03) depending on the target estimand, and for some estimands, the choice of estimator affected the conclusions by leading to smaller treatment effect estimates. We conclude that careful specification of the estimand, along with an appropriate choice of estimator, is essential to ensuring that cluster-randomised trials address the right question.

## 1 Introduction

An estimand is a precise definition of the treatment effect investigators want to estimate.^1–3^ Defining the estimand at the study's outset helps to clarify the appropriate interpretation of treatment effects and ensure that statistical methods are aligned with the study's objectives (i.e. that statistical methods are chosen to estimate the *right* treatment effect).^1–11^ Because of the clarity that estimands provide, they are becoming increasingly popular in randomised trials. The standard framework for defining an estimand requires specification of the following five attributes: (i) the population of participants; (ii) the treatment conditions; (iii) the endpoint; (iv) the summary measure (e.g. odds ratio (OR), difference, etc); and (v) how intercurrent events, such as treatment non-adherence, are handled. Importantly, the above attributes are defined in relation to the target treatment effect (e.g. the population to whom the treatment effect applies).

However, cluster-randomised trials (CRTs) (where groups of participants, such as schools or hospitals, are randomised instead of individual participants^12–18^) require the specification of additional attributes compared to individually randomised designs.^4,19^ For example, investigators must decide between *marginal* (sometimes called *population-averaged*) and *cluster-specific* (sometimes called *conditional*) treatment effects, which differ in whether outcomes are summarised overall or by cluster.^14,15,20–22^ Separately, they must also decide between *participant-average* and *cluster-average* treatment effects, which differ in how participants are weighted.^4^

Proper consideration of these attributes is important to ensure that CRTs are designed to answer the most clinically relevant question, as different estimands provide fundamentally different interpretations, and choosing the wrong estimand and/or estimator could provide misleading evidence. For instance, if interest lies in an intervention's effect across the population of participants (e.g. the number of participants that would be saved by using the intervention vs. using control), this is provided through a *participant-average* estimand. Hence, an estimator that targets a *cluster-average* estimand (such as an analysis of unweighted cluster-level summaries, which is a commonly recommended estimator in CRTs^14,15,18,23,24^), may provide a biased answer. It is therefore essential in CRTs to clearly define the estimand and then choose an appropriate estimator that targets this estimand.

The concept of marginal versus cluster-specific treatment effects has been discussed previously,^14,15,20–22^ as has the issue around how to weigh patients (i.e. participant- vs. cluster-average effects).^4,19,25–29^ However, to our knowledge, cluster-specific effects have not been formally defined using potential outcomes notation. Further, to our knowledge, these two issues have not been considered together, meaning there are currently no formal definitions for estimands which encompass both concepts nor any guidance on how these attributes differ for the construction of estimands. Finally, because these estimands have only been considered separately, the literature around estimation has also typically focused on only a single attribute of the estimand (e.g. estimation of a marginal effect, or estimation of a participant-average effect), meaning there is currently no guidance on estimation of estimands which incorporate both the marginal versus cluster-specific and participant- versus cluster-average attributes unique to CRTs.

The purpose of this paper is therefore to resolve these issues by (i) defining estimands which incorporate both attributes together (e.g. marginal, participant-average estimands; cluster-specific, participant-average estimands; etc.), and demystifying these interconnected concepts and terminology; and (ii) describing estimators that can be used for each of these estimands.

## 2 Estimands

In this section, we describe the difference between marginal and cluster-specific estimands (Section 2.2), and then separately we describe the difference between participant-average and cluster-average estimands (noting when the different estimands will coincide) (Section 2.3). A summary is provided in Table 1.

**Table 1.** Difference between marginal/cluster-specific and participant-/cluster-average attributes of estimand.

Concept	Description
**Marginal versus cluster-specific estimands**	
Marginal	A *marginal* estimand (also called ‘population-averaged’) is where the potential outcomes are first summarised separately by treatment condition, and then these summaries are contrasted between treatment conditions to obtain an overall treatment effect.
Cluster-specific	A *cluster-specific* estimand (also called ‘conditional’) is where the potential outcomes are summarised and contrasted within each cluster to obtain cluster-specific treatment effects, and then an average of these cluster-specific effects is calculated to obtain an overall treatment effect (note this average could be taken in different ways; see participant- vs. cluster-average estimands).
**Participant- versus cluster-average estimands**	
Participant-average	A *participant-average* estimand is where each participant is given equal weight
Cluster-average	A *cluster-average* estimand is where each cluster is given equal weight

We then merge the two concepts to define estimands incorporating both attributes; because there are two options for each concept, this leads to four total estimands: (i) marginal, participant-average; (ii) marginal, cluster-average; (iii) cluster-specific, participant-average; and (iv) cluster-specific, cluster-average. These are defined for both a difference in means or proportions and an OR in Section 2.4, and Table 2.

**Table 2.** Overview of estimands for cluster-randomised trials.^a^

	Super-population perspective^b^		Finite-population perspective^b^	
Estimand^c^	Definition for a different	Definition for an odds ratio	Definition for a difference^d^	Definition for an odds ratio
Marginal, participant-average ( $\Delta^{MG-PA}$ and $\Gamma^{MG-PA}$ )	$\frac{E\left(\sum_{i=1}^{n_{j}}\;Y_{ij}^{\left(1\right)}\right)}{E(n_{j})}-\frac{E\left(\sum_{i=1}^{n_{j}}\;Y_{ij}^{\left(0\right)}\right)}{E(n_{j})}$	$\frac{E\left(\sum_{i=1}^{n_{j}}\;Y_{ij}^{\left(1\right)}\right)/E(n_{j})/\left(1-E\left(\sum_{i=1}^{n_{j}}\;Y_{ij}^{\left(1\right)}\right)/E(n_{j})\right)}{E\left(\sum_{i=1}^{n_{j}}\;Y_{ij}^{\left(0\right)}\right)/E(n_{j})/\left(1-E\left(\sum_{i=1}^{n_{j}}\;Y_{ij}^{\left(0\right)}\right)/E(n_{j})\right)}$	$\frac{1}{N}\overset{M}{\underset{j=1}{\sum }}\;\overset{n_{j}}{\underset{i=1}{\sum }}\;Y_{ij}^{\left(1\right)}-\frac{1}{N}\overset{n_{J}}{\underset{j=1}{\sum }}\;Y_{j}^{\left(0\right)}$	$\frac{\frac{1}{N}\sum_{j=1}^{M}\;\sum_{i=1}^{n_{j}}\;Y_{ij}^{\left(1\right)}/\left(1-\frac{1}{N}\sum_{j=1}^{M}\;\sum_{i=1}^{n_{j}}\;Y_{ij}^{\left(1\right)}\right)}{\frac{1}{N}\sum_{j=1}^{M}\;\sum_{i=1}^{n_{j}}\;Y_{ij}^{\left(0\right)}/\left(1-\frac{1}{N}\sum_{j=1}^{M}\;\sum_{i=1}^{n_{j}}\;Y_{ij}^{\left(0\right)}\right)}$
Marginal, cluster-average ( $\Delta^{MG-CA}$ and $\Gamma^{MG-CA}$ )	$E\left(\frac{\sum_{i=1}^{n_{j}}\;Y_{ij}^{\left(1\right)}}{n_{j}}\right)-E\left(\frac{\sum_{i=1}^{n_{j}}\;Y_{ij}^{\left(0\right)}}{n_{j}}\right)$	$\frac{E\left(\sum_{i=1}^{n_{j}}\;Y_{ij}^{\left(1\right)}/n_{j}\right)/\left(1-E\left(\sum_{i=1}^{n_{j}}\;Y_{ij}^{\left(1\right)}/n_{j}\right)\right)}{E\left(\sum_{i=1}^{n_{j}}\;Y_{ij}^{\left(0\right)}/n_{j}\right)/\left(1-E\left(\sum_{i=1}^{n_{j}}\;Y_{ij}^{\left(0\right)}/n_{j}\right)\right)}$	$\frac{1}{M}\overset{M}{\underset{j=1}{\sum }}\;{{\bar{Y}}_{j}}^{\left(1\right)}-\frac{1}{M}\overset{M}{\underset{j=1}{\sum }}\;{\bar{Y}}_{j}^{\left(0\right)}$	$\frac{\frac{1}{M}\sum_{j=1}^{M}\;{{\bar{Y}}_{j}}^{\left(1\right)}/\left(1-\frac{1}{M}\sum_{j=1}^{M}\;{{\bar{Y}}_{j}}^{\left(1\right)}\right)}{\frac{1}{M}\sum_{j=1}^{M}\;{{\bar{Y}}_{j}}^{\left(0\right)}/\left(1-\frac{1}{M}\sum_{j=1}^{M}\;{{\bar{Y}}_{j}}^{\left(0\right)}\right)}$
Cluster-specific, participant-average^e^ ( $\Delta^{CS-PA}$ and $\Gamma^{CS-PA}$ )	$\frac{E(n_{j}\beta_{j})}{E(n_{j})}$	$\frac{E(n_{j}f(\text{O}{\text{R}}_{j}))}{E(n_{j})}$	$\frac{\sum_{j=1}^{M}\;n_{j}\beta_{j}}{\sum_{j=1}^{M}\;n_{j}}$	$\frac{\sum_{j=1}^{M}\;n_{j}f(\text{O}{\text{R}}_{j})}{\sum_{j=1}^{M}\;n_{j}}$
	where:	where:	where:	where:
	$\beta_{j}={{\bar{Y}}_{j}}^{\left(1\right)}-{\bar{Y}}_{j}^{\left(0\right)}$	$\text{O}{\text{R}}_{j}=\frac{\frac{1}{n_{j}}\sum_{i=1}^{n_{j}}\;Y_{ij}^{\left(1\right)}/\left(1-\frac{1}{n_{j}}\sum_{i=1}^{n_{j}}\;Y_{ij}^{\left(1\right)}\right)}{\frac{1}{n_{j}}\sum_{i=1}^{n_{j}}\;Y_{ij}^{\left(0\right)}/\left(1-\frac{1}{n_{j}}\sum_{i=1}^{n_{j}}\;Y_{ij}^{\left(0\right)}\right)}$	$\beta_{j}={{\bar{Y}}_{j}}^{\left(1\right)}-{\bar{Y}}_{j}^{\left(0\right)}$	$\text{O}{\text{R}}_{j}=\frac{\frac{1}{n_{j}}\sum_{i=1}^{n_{j}}\;Y_{ij}^{\left(1\right)}/\left(1-\frac{1}{n_{j}}\sum_{i=1}^{n_{j}}\;Y_{ij}^{\left(1\right)}\right)}{\frac{1}{n_{j}}\sum_{i=1}^{n_{j}}\;Y_{ij}^{\left(0\right)}/\left(1-\frac{1}{n_{j}}\sum_{i=1}^{n_{j}}\;Y_{ij}^{\left(0\right)}\right)}$
Cluster-specific, cluster-average^e^ ( $\Delta^{CS-CA}$ and $\Gamma^{CS-CA}$ )	$E(\beta_{j})$	$E(f(\text{O}{\text{R}}_{j}))$	$\frac{1}{M}\overset{M}{\underset{j=1}{\sum }}\;\beta_{j}$	$\frac{1}{M}\overset{M}{\underset{j=1}{\sum }}\;f(\text{O}{\text{R}}_{j})$
	where:	where:	where:	where:
	$\beta_{j}={{\bar{Y}}_{j}}^{\left(1\right)}-{\bar{Y}}_{j}^{\left(0\right)}$	$\text{O}{\text{R}}_{j}=\frac{\frac{1}{n_{j}}\sum_{i=1}^{n_{j}}\;Y_{ij}^{\left(1\right)}/\left(1-\frac{1}{n_{j}}\sum_{i=1}^{n_{j}}\;Y_{ij}^{\left(1\right)}\right)}{\frac{1}{n_{j}}\sum_{i=1}^{n_{j}}\;Y_{ij}^{\left(0\right)}/\left(1-\frac{1}{n_{j}}\sum_{i=1}^{n_{j}}\;Y_{ij}^{\left(0\right)}\right)}$	$\beta_{j}={{\bar{Y}}_{j}}^{\left(1\right)}-{\bar{Y}}_{j}^{\left(0\right)}$	$\text{O}{\text{R}}_{j}=\frac{\frac{1}{n_{j}}\sum_{i=1}^{n_{j}}\;Y_{ij}^{\left(1\right)}/\left(1-\frac{1}{n_{j}}\sum_{i=1}^{n_{j}}\;Y_{ij}^{\left(1\right)}\right)}{\frac{1}{n_{j}}\sum_{i=1}^{n_{j}}\;Y_{ij}^{\left(0\right)}/\left(1-\frac{1}{n_{j}}\sum_{i=1}^{n_{j}}\;Y_{ij}^{\left(0\right)}\right)}$

^a^*M* and *N* represent the total number of clusters and the total number of participants, respectively, $Y_{ij}^{\left(1\right)}$ and $Y_{ij}^{\left(0\right)}$ denote potential outcomes from participant *i* in cluster *j* under intervention and control, respectively, $n_{j}$ denotes the size of cluster *j*, and ${{\bar{Y}}_{j}}^{\left(1\right)}=(1/n_{j})\sum_{i=1}^{n_{j}}Y_{ij}^{\left(1\right)}$ (and similarly for ${\bar{Y}}_{j}^{\left(0\right)}$ ).

^b^Under the super-population perspective, each cluster is assumed to be an independent random sample from a hypothetical infinite population of clusters, and the expectation for each estimand is defined with respect to that super-population of clusters. Under the finite-population perspective, the trial sample itself is considered as the finite target population, and so the estimand is defined for these clusters. Mathematically, the difference between the two frameworks is that one uses population expectation for the estimand while the other considers a finite-sample estimand.

^c^In order to be fully defined, estimands require further specification of the (i) population; (ii) treatment conditions; (iii) endpoint; (iv) summary measure; and (v) handling of intercurrent events.

^d^For differences, marginal and cluster-specific estimands coincide.

^e^ $f(\text{O}{\text{R}}_{j})$ is some function of the cluster-specific odds ratio, for instance, identity or log. Each function corresponds to a different way of averaging the cluster-specific odds ratios across clusters. For instance, an identity function, where $f(\text{O}{\text{R}}_{j})=\text{O}{\text{R}}_{j}$ would just take a simple average of the odds ratios. A log function, where $f(\text{O}{\text{R}}_{j})=\log\;(\text{O}{\text{R}}_{j})$ would take the average of the log-odds ratios (importantly, the resulting average would need to be back-transformed to the odds ratio scale, e.g. by taking $\exp\;\left[\sum_{j=1}^{M}\;n_{j}f(\text{O}{\text{R}}_{j})/\sum_{j=1}^{M}\;n_{j}\right]$ or $\text{exp}\left[(1/M)\sum_{j=1}^{M}f(\text{O}{\text{R}}_{j})\right]$ . We recommend using $f(\text{O}{\text{R}}_{j})=\log\;(\text{O}{\text{R}}_{j})$ as this typically leads to more interpretable results; see Sections 2.4.3 and 2.4.4.

We note that to be fully defined, each of the estimands described below would require specification of the other attributes encompassing an estimand (i.e. population, treatment conditions, endpoint, summary measure, and handling of intercurrent events^1,30^). Some of these attributes may require additional consideration in CRTs; for instance, the population of interest would need to be described for clusters as well as participants, and investigators may need to differentiate whether interest lies in the population of all eligible participants versus just those that would enrol in the study if provided the opportunity.^31,32^ Similarly, in some CRTs, investigators may need to describe the duration of the implementation of the intervention (e.g. the average effect over three vs. six months of implementation^33,34^) when describing the treatment conditions of interest. Similarly, because non-adherence could occur both at the participant or cluster level, they may need to define intercurrent events at both the individual and cluster levels. To our knowledge, there are unlikely to be any additional considerations in CRTs when defining the endpoint or summary measure attributes (apart from the cluster-specific vs. marginal distinction discussed in this paper). To facilitate a clearer description of our main message, we do not further address these additional considerations below.

We describe each estimand using the potential outcomes framework. We do this under two different perspectives that have been used for defining causal effects in CRTs: (i) a super-population perspective, where each cluster is assumed to be an independent random sample from a hypothetical infinite population of clusters^26,29,35^; and (ii) a finite-population perspective, where the clusters in the trial are themselves considered as the target population.^28^ The key difference between these is that under the super-population perspective, the estimand is written in terms of a population expectation (taken over the infinite population of clusters), while under the finite-population perspective, the estimand is written as an empirical average across the observed clusters and participants in the study. Though the concepts behind the participant- versus cluster-average and marginal versus cluster-specific aspects are the same under each perspective, we provide both for completeness. For simplicity, however, we focus on describing the estimands under the finite-population perspective in what follows and include those under the super-population perspective in Table 1.

### 2.1 Notation

We begin by introducing the notation that will be used to define both the estimands and the estimators below. Let $Y_{ij}$ denote the observed outcome for participant *i* from cluster *j*, and $Z_{j}$ represent the treatment assignment for cluster *j*. Further, let $n_{j}$ be the number of participants in cluster *j*, and *M* and *N* represent the total number of clusters and the total number of participants, respectively.

Under the potential outcome framework, $Y_{ij}^{\left(1\right)}$ denotes the potential outcome that would have been realised under $Z_{j}=1$ for participant *i* from cluster *j*, and similarly $Y_{ij}^{\left(0\right)}$ represents that participant's potential outcome under $Z_{j}=0$ . Then, let ${\bar{Y}}_{j}^{\left(1\right)}$ denote the average potential outcome in cluster *j* under treatment $Z_{j}=1$ , that is,

(1) $${\bar{Y}}_{j}^{\left(1\right)}=\frac{1}{n_{j}}\overset{n_{j}}{\underset{i=1}{\sum }}\;Y_{ij}^{\left(1\right)}$$

and similarly for ${\bar{Y}}_{j}^{\left(0\right)}$ under the control condition.

### 2.2 Marginal and cluster-specific estimands

We provide formal definitions for marginal and cluster-specific estimands below. Briefly, the difference between marginal and cluster-specific estimands is based on how the potential outcomes are summarised.

A marginal estimand is calculated using the following steps:

1) Overall summaries are obtained by summarising the potential outcomes separately by treatment condition using all participants (e.g. the mean potential outcome is calculated under the intervention and control, respectively).
2) The summaries are contrasted between treatment conditions to calculate an overall marginal treatment effect.

Conversely, for a cluster-specific estimand, the following steps are taken:

1) Cluster-specific summaries are obtained by summarising the potential outcomes *within each cluster* (e.g. the mean potential outcome is calculated under intervention and control, respectively, in cluster 1, cluster 2, etc.).
2) The cluster-specific summaries are contrasted between treatment conditions within each cluster to calculate a cluster-specific treatment effect.
3) An average of these cluster-specific effects is calculated to provide the overall cluster-specific estimand (note this average can be taken in different ways; see Section 2.3. on participant- vs. cluster-average estimands below).

Thus, the difference between marginal and cluster-specific estimands is whether an overall summary measure is calculated within each treatment arm before the arms are contrasted (marginal estimand), or whether summary measures are contrasted within each cluster first (cluster-specific estimand).

For certain summary measures, the overall cluster-specific estimand may average over some function of the cluster-specific effects. For instance, when defining an estimand based on an OR, the average of the log OR may be taken across clusters, then back-transformed after to obtain the overall cluster-specific OR. This example will be further discussed in Section 2.4.

We note that marginal and cluster-specific estimands can be written as either participant-average or cluster-average treatment effects (depending on how each individual or cluster will be weighted, described in Section 2.3); for simplicity, we describe the differences between marginal and cluster-specific estimands below in terms of participant-average effects.

#### 2.2.1 Marginal estimands

For a difference in means or proportions, a marginal participant-average estimand is defined under the finite-population perspective as:

(2) $$\Delta^{MG-PA}=\frac{1}{N}\overset{M}{\underset{j=1}{\sum }}\;\overset{n_{j}}{\underset{i=1}{\sum }}\;Y_{ij}^{\left(1\right)}-\frac{1}{N}\overset{M}{\underset{j=1}{\sum }}\;\overset{n_{j}}{\underset{i=1}{\sum }}\;Y_{ij}^{\left(0\right)}$$

#### 2.2.2 Cluster-specific estimands

The cluster-specific estimand for a difference in means/proportions can be defined under the finite-population perspective as follows. First, let $\beta_{j}$ represent the cluster-specific treatment effect for cluster *j*, that is,

(3) $$\beta_{j}={{\bar{Y}}_{j}}^{\left(1\right)}-{\bar{Y}}_{j}^{\left(0\right)}$$

where ${{\bar{Y}}_{j}}^{\left(1\right)}$ and ${\bar{Y}}_{j}^{\left(0\right)}$ are defined based on equation (1). Then, the cluster-specific participant-average estimand is defined based on a weighted linear combination of the $\beta_{j}\text{s}$ :

(4) $$\Delta^{CS-PA}=\frac{\sum_{j=1}^{M}\;n_{j}\beta_{j}}{\sum_{j=1}^{M}\;n_{j}}=\frac{1}{N}\overset{M}{\underset{j=1}{\sum }}\;\overset{n_{j}}{\underset{i=1}{\sum }}\;Y_{ij}^{\left(1\right)}-\frac{1}{N}\overset{M}{\underset{j=1}{\sum }}\;\overset{n_{j}}{\underset{i=1}{\sum }}\;Y_{ij}^{\left(0\right)}$$

#### 2.2.3 When will marginal and cluster-specific estimands coincide or differ?

We use the term *collapsible* to indicate that the values of the two estimands will coincide, and *non-collapsible* to mean that the values of the two estimands will differ.^36^

Whether the marginal and cluster-specific estimands will coincide or not depends on the summary measure (e.g. difference and OR) being used. For differences (e.g. difference in means and difference in proportions), these two estimands will coincide (i.e. $\Delta^{MG-PA}=\Delta^{CS-PA}$ ) because the empirical average is a linear operator. Specifically, we can see from equation (4) that it is mathematically equivalent to first summarise outcomes overall with an empirical average and then take a difference, or to take a difference within clusters and then take an average of these differences. Because the two estimands are the same, a ‘difference’ summary measure is *collapsible*.

For ratio summary measures (e.g. risk ratios and ORs), in general, the two estimands will differ. This is because of the function used which transforms the summaries (e.g. by taking the log or logit transformation of the marginal or cluster-specific summaries); this feature renders the mathematical equivalency stated above invalid; that is, a ratio of overall summaries is generally *not* the same as an average of the ratios within each cluster (except in a few specific settings, e.g. if the risk ratio is identical across all clusters). Because the marginal and cluster-specific estimands are different, ‘ratio’ summary measures are *non-collapsible*, except in special circumstances.

### 2.3 Participant- and cluster-average estimands

We provide formal definitions for participant- and cluster-average estimands under the finite-population perspective below. Briefly, the difference between the participant- and cluster-average estimands is in how the potential outcomes are weighted.^4^ Under the participant-average estimand, a general principle is that each participant is given equal weight. Under the cluster-average effect definition, a general principle is that each cluster is given equal weight (implying that participants from smaller clusters are given more weight than participants from larger clusters).^4^

We note that both the participant- and cluster-average treatment effects can be written as either marginal or cluster-specific estimands; however, in this section, we write them as marginal estimands for simplicity.

#### 2.3.1 Participant-average estimands

For a difference in means, or difference in proportions, the marginal participant-average estimand is given in equation (2) above (as we defined marginal estimands above as participant-average for simplicity). We repeat this equation here for completeness:

$$\Delta^{MG-PA}=\frac{1}{N}\overset{M}{\underset{j=1}{\sum }}\;\overset{n_{j}}{\underset{i=1}{\sum }}\;Y_{ij}^{\left(1\right)}-\frac{1}{N}\overset{M}{\underset{j=1}{\sum }}\;\overset{n_{j}}{\underset{i=1}{\sum }}\;Y_{ij}^{\left(0\right)}$$

To provide additional insights into this definition, an alternative representation of the marginal participant-average estimand is^28^:

$$\Delta^{MG-PA}=\frac{1}{M}\overset{M}{\underset{j=1}{\sum }}\;\frac{n_{j}M}{N}{{\bar{Y}}_{j}}^{\left(1\right)}-\frac{1}{M}\overset{M}{\underset{j=1}{\sum }}\;\frac{n_{j}M}{N}{\bar{Y}}_{j}^{\left(0\right)}$$

which reveals that the weight of each cluster-specific summary measure ( ${{\bar{Y}}_{j}}^{\left(1\right)}$ and ${\bar{Y}}_{j}^{\left(0\right)}$ , which are defined based on equation (1)) is proportional to its cluster size $n_{j}$ , implying that a larger cluster is given more weight than a smaller cluster. This representation is helpful as it underlies a key distinction between participant- and cluster-average estimands.

#### 2.3.2 Cluster-average estimands

For a difference in means/proportions, the marginal cluster-average estimand is defined as:

(5) $$\Delta^{MG-CA}=\frac{1}{M}\overset{M}{\underset{j=1}{\sum }}\;{{\bar{Y}}_{j}}^{\left(1\right)}-\frac{1}{M}\overset{M}{\underset{j=1}{\sum }}\;{\bar{Y}}_{j}^{\left(0\right)}$$

As above, to provide additional insights into this definition, we provide an alternative representation of the marginal cluster-average estimand:

$$\Delta^{MG-CA}=\frac{1}{N}\overset{M}{\underset{j=1}{\sum }}\;\left(\frac{1}{n_{j}}\overset{n_{j}}{\underset{i=1}{\sum }}\;Y_{ij}^{\left(1\right)}\right)-\frac{1}{N}\overset{M}{\underset{j=1}{\sum }}\;\left(\frac{1}{n_{j}}\overset{n_{j}}{\underset{i=1}{\sum }}\;Y_{ij}^{\left(0\right)}\right)$$

This clearly demonstrates that cluster-average estimands give equal weight to each cluster, regardless of the cluster size used to generate the summaries ${{\bar{Y}}_{j}}^{\left(1\right)}$ and ${\bar{Y}}_{j}^{\left(0\right)}$ .

#### 2.3.3 When will participant- and cluster-average estimands coincide or differ?

Whether the participant- and cluster-average estimands will differ depends on two things: (i) whether the estimand summary measure being used is *collapsible* (e.g. a difference in means or proportions) or *non-collapsible* (e.g. a ratio, except in specific circumstances); and (ii) whether informative cluster size is present. Informative cluster size occurs when either the potential outcomes or potential outcome contrasts (i.e. $Y_{ij}^{\left(1\right)}-Y_{ij}^{\left(0\right)}$ for a difference in means or proportions) depend on cluster size.^37,38^

For collapsible summary measures, such as the difference in means/proportions, the participant- and cluster-average estimands will differ when the second type of informative cluster size is present, that is, when the potential outcome contrasts differ according to cluster size.^4^

For non-collapsible summary measures, such as the OR, the participant- and cluster-average treatment effect will typically differ when either the first or second type of informative cluster size is present, that is, when either the outcomes or the treatment effects differ depending on the cluster size.^4^

### 2.4 Estimands encompassing both attributes (marginal vs. cluster-specific attribute and participant- vs. cluster-average attribute)

Estimand definitions incorporating both attributes (marginal vs. cluster-specific attribute and participant- vs. cluster-average attribute) are described below and summarised in Table 2. Because there are two options for each attribute, this leads to four total estimands. Below we describe the construction of these estimands for both a difference and an OR summary measure.

#### 2.4.1 Marginal participant-average estimand

The marginal participant-average estimand for a difference is given in equation (2) above, and we repeat it here for convenience:

$$\Delta^{MG-PA}=\frac{1}{N}\overset{M}{\underset{j=1}{\sum }}\;\overset{n_{j}}{\underset{i=1}{\sum }}\;Y_{ij}^{\left(1\right)}-\frac{1}{N}\overset{M}{\underset{j=1}{\sum }}\;\overset{n_{j}}{\underset{i=1}{\sum }}\;Y_{ij}^{\left(0\right)}$$

Similarly, the marginal participant-average OR (we use the notation Γ to denote the OR estimands to differentiate from the difference in means notation Δ ) for binary outcomes can be defined as:

(6) $$\Gamma^{MG-PA}=\frac{(1/N)\sum_{j=1}^{M}\;\sum_{i=1}^{n_{j}}\;Y_{ij}^{\left(1\right)}/\left(1-(1/N)\sum_{j=1}^{M}\;\sum_{i=1}^{n_{j}}\;Y_{ij}^{\left(1\right)}\right)}{(1/N)\sum_{j=1}^{M}\;\sum_{i=1}^{n_{j}}\;Y_{ij}^{\left(0\right)}/\left(1-(1/N)\sum_{j=1}^{M}\;\sum_{i=1}^{n_{j}}\;Y_{ij}^{\left(0\right)}\right)}$$

#### 2.4.2 Marginal cluster-average estimand

For a difference in means/proportions, the marginal cluster-average estimand is given in equation (5); we repeat it here for convenience:

$$\Delta^{MG-CA}=\frac{1}{M}\overset{M}{\underset{j=1}{\sum }}\;{{\bar{Y}}_{j}}^{\left(1\right)}-\frac{1}{M}\overset{M}{\underset{j=1}{\sum }}\;{\bar{Y}}_{j}^{\left(0\right)}$$

And the marginal cluster-average OR for binary outcomes is:

(7) $$\Gamma^{MG-CA}=\frac{(1/M)\sum_{j=1}^{M}\;{{\bar{Y}}_{j}}^{\left(1\right)}/\left(1-(1/M)\sum_{j=1}^{M}\;{{\bar{Y}}_{j}}^{\left(1\right)}\right)}{(1/M)\sum_{j=1}^{M}\;{{\bar{Y}}_{j}}^{\left(0\right)}/\left(1-(1/M)\sum_{j=1}^{M}\;{{\bar{Y}}_{j}}^{\left(0\right)}\right)}$$

where definitions of ${{\bar{Y}}_{j}}^{\left(1\right)}$ and ${\bar{Y}}_{j}^{\left(0\right)}$ are given in equation (1). In other words, this estimand is constructed by applying the OR summary measure to the cluster-average potential outcomes, $(1/M)\sum_{j=1}^{M}{{\bar{Y}}_{j}}^{\left(1\right)}$ and $(1/M)\sum_{j=1}^{M}{{\bar{Y}}_{j}}^{\left(0\right)}$ .

#### 2.4.3 Cluster-specific, participant-average estimand

The cluster-specific participant-average estimand for a difference in means/proportions is given in equation (4); we repeat it here for convenience:

$$\Delta^{CS-PA}=\frac{\sum_{j=1}^{M}\;n_{j}\beta_{j}}{\sum_{j=1}^{M}\;n_{j}}$$

where $\beta_{j}$ was defined in equation (3). For a difference in means/proportions, $\Delta^{CS-PA}=\Delta^{MG-PA}$ due to collapsibility.

For the cluster-specific participant-average OR, one can first let $\text{O}{\text{R}}_{j}$ denote the cluster-specific OR for cluster *j*, that is:

(8) $${\text{OR}}_{j}=\frac{(1/n_{j})\sum_{i=1}^{n_{j}}\;Y_{ij}^{\left(1\right)}/\left(1-(1/n_{j})\sum_{i=1}^{n_{j}}\;Y_{ij}^{\left(1\right)}\right)}{(1/n_{j})\sum_{i=1}^{n_{j}}\;Y_{ij}^{\left(0\right)}/\left(1-(1/n_{j})\sum_{i=1}^{n_{j}}\;Y_{ij}^{\left(0\right)}\right)}$$

Then, the cluster-specific participant-average effect can be defined based on a cluster size weighted average as:

(9) $$\frac{\sum_{j=1}^{M}\;n_{j}f(\text{O}{\text{R}}_{j})}{\sum_{j=1}^{M}\;n_{j}}$$

where $f(\text{O}{\text{R}}_{j})$ is some function of the cluster-specific OR, for instance, the identity or log function. Each function corresponds to a different way of averaging the cluster-specific ORs across clusters. Typically, a log function, where $f(\text{O}{\text{R}}_{j})=\log\;(\text{O}{\text{R}}_{j})$ , would be most natural for ORs. This is because (i) the log OR is connected to regression parameters with a canonical link function and is a familiar concept, and (ii) the resulting average in equation (9) can be interpreted as a cluster size weighted geometric mean of $\text{O}{\text{R}}_{j}$ , that is,

$$\log\;\left\{{\left(\overset{M}{\underset{j=1}{\prod }}\;{\text{OR}}_{j}^{n_{j}}\right)}^{1/\sum_{j=1}^{M}\;n_{j}}\right\}$$

Importantly, when a function other than identity is used, the resulting average may need to be back-transformed to the appropriate scale. For example, when *f* is a log function, a back transformation should be applied to equation (9) to obtain the final cluster-specific, participant-average estimand, that is,

$$\Gamma^{CS-PA}=\exp\;\left(\frac{\sum_{j=1}^{M}\;n_{j}\log\;(\text{O}{\text{R}}_{j})}{\sum_{j=1}^{M}\;n_{j}}\right)$$

Of note, the estimand in (9) can be seen to correspond to a participant-average effect because each participant gets equal weight in the construction of the cluster-specific contrasts of potential outcomes (the $\text{O}{\text{R}}_{j}\text{s}$ in equation (8)), and each cluster-specific contrast is weighted according to the number of participants belonging to that cluster.

In general, cluster-specific estimands (participant- or cluster-average) are only well defined when the cluster-specific treatment effects are well defined, for example, for an OR this would require the potential outcome proportion to be bounded away from 0 or 1 in each cluster so that a cluster-specific ORs from equation (8) can be defined without ambiguity.

#### 2.4.4 Cluster-specific, cluster-average estimand

The cluster-specific cluster-average estimand for a difference in means/proportions is defined as:

(10) $$\Delta^{CS-CA}=\frac{1}{M}\overset{M}{\underset{j=1}{\sum }}\;\beta_{j}$$

where $\beta_{j}$ was defined in equation (3). For a difference in means, $\Delta^{CS-CA}=\Delta^{MG-CA}$ due to collapsibility.

The cluster-specific cluster-average OR can be defined by giving equal weight to each cluster-specific summary:

(11) $$\frac{1}{M}\overset{M}{\underset{j=1}{\sum }}\;f(\text{O}{\text{R}}_{j})$$

where $\text{O}{\text{R}}_{j}$ was defined in equation (8), and $f(\text{O}{\text{R}}_{j})$ was defined as in Section 2.4.3. When *f* is a log function, equation (11) is interpreted as the geometric mean of $\text{O}{\text{R}}_{j}$ as $\log\;[{\left(\prod_{j=1}^{M}\text{O}{\text{R}}_{j}\right)}^{1/M}]$ . In this case, a final back transformation should be applied to equation (11) to define the final cluster-specific, cluster-average estimand as:

$$\Gamma^{CS-CA}=\exp\;\left(\frac{1}{M}\overset{M}{\underset{j=1}{\sum }}\;\log\;(\text{O}{\text{R}}_{j})\right)$$

## 3 Estimators

We now describe some familiar estimators that can be used to estimate each of the estimands described previously for a parallel-arm CRT. We focus on the following estimators: (a) independence estimating equations (IEEs)^4,19^; (b) the analysis of cluster-level summaries^4,15,18^; (c) mixed-effects models with a cluster-level random intercept; and generalised estimating equations (GEEs) with an exchangeable working correlation structure. We focus on the simple scenario without baseline covariate adjustment for each estimand. When covariate adjustment is of interest to obtain more efficient estimators, we refer readers to Su and Ding^28^ for related development under a finite-population perspective, Balzer et al.,^39,40^ Benitez et al.,^25^ and Wang et al.^29^ for related developments under a super-population perspective; although these prior developments often focus only on a subset of estimands we have covered. Below, we address commonly used estimators for CRTs in the absence of covariate adjustment, and then explain which estimands they target, along with the key assumptions required for consistency (i.e. asymptotically unbiased estimation). A summary is given in Table 3.

**Table 3.** Overview of estimators for each estimand.

Estimand	Description	Estimator	Description	Assumptions
Marginal, participant-average	Average effect across participants. Potential outcomes are summarised under each treatment condition, and these summaries are contrasted.	IEEs (unweighted)	Individual participant outcomes are analysed using an independent working correlation structure in conjunction with cluster-robust standard errors. Can be implemented using (a) GEEs with a working independence correlation structure or (b) using maximum likelihood/least squares estimators (both options (a) and (b), using cluster-robust standard errors); importantly, the models are unweighted.	Standard assumptions^a^
		Cluster-level summaries (weighted)	A summary measure is taken in each cluster (e.g. the proportion), and an appropriate weighted generalised linear model is used for analysis, with the cluster-level summaries as the outcomes, and weights equal to the cluster size $n_{j}$ .^b^ For instance, a logit link could be used for the odds ratio; a family should be chosen such that the variance is independent of the mean (e.g. Gaussian).	Standard assumptions^a^
		GEEs with exchangeable correlation (unweighted)	GEEs are applied to individual participant data, using an exchangeable working correlation structure, in conjunction with cluster-robust standard errors.	Standard assumptions^a^ Non-informative cluster size
Marginal, cluster-average	Average effect across clusters Potential outcomes are summarised under each treatment condition, and these summaries are contrasted	IEEs (weighted)	As described above (under ‘Marginal, participant average’ estimand); however, participants are weighted by their inverse cluster size, $1/n_{j}$ .^b^	Standard assumptions^a^
		Cluster-level summaries (unweighted)	As described above (under ‘Marginal, participant average’ estimand); however, the generalised linear model is unweighted.	Standard assumptions^a^
Cluster-specific, participant-average	Average effect across participants Potential outcomes are summarised and contrasted within each cluster separately, and these cluster-specific effects are then averaged, giving equal weight to each participant	Cluster-level summaries (weighted)	First, a summary measure is taken in each cluster (e.g. a proportion). Next, the summary measure is transformed if necessary; for instance, if the aim is to estimate an odds ratio, the log odds are taken for each cluster. Finally, a weighted linear regression model is applied to the cluster-specific (transformed) summary measures, with weights equal to the cluster size $n_{j}$ .^b^ The estimate may then need to be back-transformed (e.g. if the log odds have been used, the odds ratio is calculated by exponentiating the estimate).	Standard assumptions^a^ For binary outcomes, the average outcome in each cluster is bounded away from 0 or 1
		Mixed-effects model	A mixed-effects model is applied to individual participant data, with a random intercept for cluster.	Standard assumptions^a^ Non-informative cluster size
Cluster-specific, cluster-average	Average effect across clusters Potential outcomes are summarised and contrasted within each cluster separately, and these cluster-specific effects are then averaged, giving equal weight to each cluster	Cluster-level summaries (unweighted)	As described above (under ‘Cluster-specific, participant average’ estimand); however, the regression model is unweighted.	Standard assumptions.^a^ For binary outcomes, the average outcome in each cluster is bounded away from 0 or 1.

GEE: generalised estimating equation; IEE: independence estimating equation.

^a^Standard assumptions are: (a) the consistency assumption (sometimes termed the stable unit treatment value assumption), which requires that $Y_{ij}=Y_{ij}^{\left(z\right)}$ for Z=z , that is, that a participant's observed outcome is equal to their potential outcome under their allocated treatment; (b) the maximum cluster size is bounded; (c) exchangeability between treatment arms (i.e. that clusters are randomised between treatment arms, and there is no differential enrolment of patients between treatments); (d) observations are independent across clusters; and (e) a large number of clusters such that appropriate versions of Law of Large Numbers and Central Limit Theorems can be applied.

^b^The cluster size $n_{j}$ is based on the number of participants in each cluster included in the analysis.

In general, the assumptions required to consistently estimate the target estimand are similar under both perspectives (super-population vs. finite-population), except for some technical differences in conceptualising the asymptotic regime and versions of the Central Limit Theorems invoked^41,42^; for simplicity, we do not distinguish between these two perspectives and only discuss the necessary assumptions with easy-to-understand terms.

We note that this list of estimators we describe is not intended to be comprehensive. In particular, we focus on the standard implementations of mixed-effects models and GEEs with an exchangeable correlation structure, in which the estimated treatment effect is taken from the model parameter corresponding to the assigned treatment. However, there are other implementations that could be used (e.g. model-based g-computation estimators based on linear-mixed models and GEEs in Section 3 in Wang et al.,^29^ and propensity score weighting estimators in Zhu et al.^43^), which can consistently estimate the participant-average and cluster-average estimands even when the associated working models are misspecified.

In general, all estimators described above require the following assumptions: (i) the consistency assumption (sometimes termed the cluster-level stable unit treatment value assumption), which requires that $Y_{ij}=Y_{ij}^{\left(z\right)}$ for Z=z , that is, that a participant's observed outcome is equal to their potential outcome under their allocated treatment and is defined without ambiguity^44^; (ii) the maximum cluster size is bounded; (iii) exchangeability between treatment arms^44^ (i.e. that clusters are randomised between treatment arms, and there is no differential enrolment of patients between treatments^31,32^); (iv) observations are independent across clusters^29^; (v) a large number of clusters such that appropriate versions of Law of Large Numbers and Central Limit Theorems can be applied.

In addition, estimators for cluster-specific estimands with a binary outcome will also require the assumption that the average potential outcome in each cluster is bounded away from 0 or 1. We discuss additional assumptions required for mixed-effects models and GEEs with an exchangeable correlation structure below.

Of note, we slightly abuse the notation throughout this section such that β represents the treatment regression coefficient across different working models. We caution that the regression coefficient should be interpreted based on each working model separately and not compared across models.

### 3.1 Independence estimating equations (IEEs)

IEEs are a class of estimators which is applied to individual participant outcomes and use an independent working correlation structure in conjunction with cluster-robust standard errors (SEs).^45^ Briefly, IEEs make a working assumption that outcomes within a cluster are independent; in practice, this assumption will almost always be false for CRTs; however, it helps ensure consistent (asymptotically unbiased) estimation in the presence of informative cluster size.^4^ The cluster-robust SEs then serve to ensure estimated SEs are asymptotically valid despite the incorrect working independence assumption.^46^

IEEs can be used to estimate marginal, participant-average effects, as well as marginal, cluster-average effects (using different specifications of weights). However, they cannot be used to estimate cluster-specific effects. They do not require any assumptions beyond those specified earlier.

#### 3.1.1 Marginal, participant-average estimator

For a difference in means, IEEs can be implemented to estimate the marginal, participant-average effect by applying the following model to individual participant data:

(12) $$Y_{ij}=\alpha +\beta Z_{j}+\varepsilon_{ij}$$

In this model, an independent working correlation structure and a constant variance structure are specified for $\varepsilon_{ij}$ . Estimation of β can then be done either using a linear regression model (which makes the working independence assumption automatically) or by using GEEs with a working independence correlation structure alongside an ‘identity’ link and ‘Gaussian’ family. Importantly, for both approaches (linear regression, GEEs) cluster-robust SEs must be specified to account for correlation between outcomes within the same cluster.^28,45^

Similarly, the marginal, participant-average OR can be estimated through the following model:

(13) $$\mathrm{logit}(P\{Y_{ij}=1\})=\alpha +\beta Z_{j}$$

As above, this model also uses an independent working correlation structure and could be applied either using a standard logistic regression model or GEEs with a working independent correlation structure alongside a ‘logit’ link and ‘binomial’ family (making sure to use cluster-robust SEs in both cases).^43^ Of note, to estimate the final marginal, participant-average OR, one needs to exponentiate the treatment effect coefficient in (13), that is, $\Gamma^{MG-PA}=\exp\;(\beta )$ ; a proof of consistency under the super-population perspective can be found in Web Appendix 1 of Zhu et al.^43^

Because the models in equations (12) and (13) give equal weight to each participant, they correspond to a participant-average effect, and because the models first summarise outcomes within treatment groups before applying any transformations, they correspond to marginal effects.

#### 3.1.2 Marginal, cluster-average estimator

IEEs can be used to estimate a marginal, cluster-average effect using models (12) and (13) above; however, each individual observation is additionally weighted by the inverse cluster size $1/n_{j}$ . This is to ensure each cluster is given an equal weight of 1 (i.e. weighting by $1/n_{j}$ ensures the sum of weights across participants in each cluster is equal to 1). As above, an independent working correlation structure is used alongside cluster-robust SEs.

Because these models give equal weight to each cluster, they correspond to a cluster-average effect. As above, because they summarise outcomes within treatment groups before applying any transformations, they correspond to marginal effects. In the case of an OR summary measure, a simple modification of the proof in Web Appendix 1 of Zhu et al.^43^ can be used to show that this weighted IEE estimator is consistent.

### 3.2 Analysis of cluster-level summaries

The analysis of cluster-level summaries involves two steps: (i) a summary measure is taken in each cluster (e.g. the mean observed outcome across all participants in the cluster) and (ii) the analysis is performed on the cluster-level summaries.

The analysis of cluster-level summaries can be used to estimate all four estimands described previously (marginal, participant-average effects; marginal, cluster-average effects; cluster-specific participant-average effects; and cluster-specific, cluster-average effects). For illustration, we describe the different implementations below for an OR summary measure (we note that for a difference in means/proportions, implementations of marginal and cluster-specific estimators are identical; this is because no transformation of the marginal/cluster-specific summaries needs to be taken). They require the standard assumptions specified earlier. Further, the cluster-specific estimators require the same assumption as required for the cluster-specific estimand, that is, that the potential outcome proportions need to be bounded away from 0 or 1 in each cluster so that an OR summary within each cluster is well defined. We provide proof of consistency (i.e. asymptotically unbiased estimation) for each cluster-level summary approach described below in the Appendix.

#### 3.2.1 Marginal, participant-average estimator

This estimator uses a two-step procedure. In the first step, the proportion of events in each cluster is calculated, represented by ${\hat{\pi }}_{j}$ for cluster *j*. Then, a weighted generalised linear model (GLM) using an appropriate link function (logit for an OR) is applied using the ${\hat{\pi }}_{j}\text{s}$ as outcomes, that is:

(14) $$\text{logit}(E({\hat{\pi }}_{j}))=\alpha +\beta Z_{j}$$

and during estimation, each cluster-level summary is weighted by $n_{j}$ (to give equal weight to each participant). To implement the GLM, a working distribution family must also be chosen (e.g. binomial, Gaussian, etc). We show in Section A.1 that different choices of distribution family for the model in (14) have no impact on the estimation of β ; this is a special result since the model has no additional covariates beyond the treatment indicator. For simplicity, we use a ‘Gaussian’ family, which is consistent with the standard implementation of a cluster-level summaries approach (in which the summaries are compared in a linear regression model; this is described below). The final OR parameter is calculated by $\Gamma^{MG-PA}=\exp\;(\beta )$ .

Because this model gives equal weight to each participant, it corresponds to a participant-average effect. Furthermore, because this model summarises outcomes within the treatment group before applying any transformations, it estimates a marginal effect.

#### 3.2.2 Marginal, cluster-average estimator

Cluster-level summaries can be used to estimate a marginal, cluster-average effect, using model (14) above; however, the cluster-level summaries are unweighted in order to give equal weight to each cluster. The final OR parameter is calculated by $\Gamma^{MG-CA}=\exp\;(\beta )$ . As above, in addition to showing the consistency of this estimator, in Section A.2 we demonstrate that the choice of distribution family has no impact on the estimation of β .

#### 3.2.3 Cluster-specific, participant-average estimator

This estimator uses a three-step procedure, as follows:

1. As above, the proportion of observed events in each cluster ( ${\hat{\pi }}_{j}$ ) is calculated.
2. The proportions from Step 1 are transformed according to the summary measure being used; for instance, an OR would require calculating the log odds in each cluster, that is, $\log\;(\text{odd}{\text{s}}_{j})=\log\;[{\hat{\pi }}_{j}/(1-{\hat{\pi }}_{j})]$ .
3. Finally, the cluster-level summaries calculated in step 2 (e.g. the $\log\;(\text{odd}{\text{s}}_{j})=\log\;[{\hat{\pi }}_{j}/(1-{\hat{\pi }}_{j})]$ ) are analysed using model (15) below, which is a weighted linear regression working model (where each cluster-level summary is weighted by $n_{j}$ , and the treatment indicator is the independent variable).
(15) $$\text{log}(\text{odd}{\text{s}}_{j})=\alpha +\beta Z_{j}+\varepsilon_{j}$$
  where $\text{log}(\text{odd}{\text{s}}_{j})$ was defined in step 2. Note that this model could be equivalently written as:
$$\text{logit}({\hat{\pi }}_{j})=\alpha +\beta Z_{j}+\varepsilon_{j}\;$$

The treatment effect estimate is then back-transformed as appropriate (e.g. the OR is then calculated as $\Gamma^{CS-PA}=\exp\;(\beta )$ ). As discussed above, for a difference in means/proportions, no transformation is required (i.e. the cluster-specific and marginal estimators are identical).

Because this estimation procedure weights each cluster-specific summary by the cluster size (hence intuitively giving each participant the same weight), it corresponds to a participant-average effect. Furthermore, because it applies transformations to the cluster-level summaries directly (rather than summarising the entire treatment arm before applying the transformation) it targets a cluster-specific effect. As above, we sketch the proof for consistency in Section A.3.

#### 3.2.4 Cluster-specific, cluster-average estimator

Cluster-level summaries can be used to estimate a cluster-specific, cluster-average effect, using model (15) above; however, the cluster-level summaries are unweighted in order to give equal weight to each cluster. A proof of consistency is given in Section A.4.

### 3.3 Mixed-effects models

Mixed-effects models are applied to participant level data and involve specifying a random intercept term for the clusters. For a difference in means, a linear mixed-effects model takes the form:

(16) $$Y_{ij}=\alpha +\beta Z_{j}+\mu_{j}+\varepsilon_{ij}$$

where $\mu_{j}$ represents a random effect for cluster *j*, which is assumed to follow a normal distribution with mean 0 and variance $\sigma_{B}^{2}$ , and $\varepsilon_{ij}$ is a random error term for participant *i* from cluster *j*, which is assumed to follow a normal distribution with mean 0 and variance $\sigma_{W}^{2}$ . Estimation is performed using maximum likelihood (or restricted maximum likelihood).

For estimating an OR, a logistic mixed-effects model with a random intercept is:

(17) $$\mathrm{logit}(P\{Y_{ij}=1\})=\alpha +\beta Z_{j}+\mu_{j}$$

Mixed-effects models have been conventionally considered as tools to estimate a cluster-specific, participant-average effect. However, by construction of the estimators, they do not in fact give equal weight to each participant (or, equivalently, weight each cluster by its respective cluster size). In fact, Wang et al.^19^ have shown that the generalised least squares estimator of β in model (16) targets the following quantity (expressed under a finite-population perspective):

$$\Delta (\rho *)=\frac{\sum_{j=1}^{M}\;\frac{n_{j}}{1+(n_{j}-1)\rho *}\{{{\bar{Y}}_{j}}^{\left(1\right)}-{{\bar{Y}}_{j}}^{\left(0\right)}\}}{\sum_{j=1}^{M}\;\frac{n_{j}}{1+(n_{j}-1)\rho *}}$$

where ρ* is the probability limit of the intracluster correlation estimator, ${\hat{\sigma }}_{B}^{2}/({\hat{\sigma }}_{B}^{2}+{\hat{\sigma }}_{W}^{2})$ . Thus, clusters are weighted by their inverse-variance (also referred to as the precision weights), with weights additionally depending on the unknown variance components.^4^ While this weighting scheme is motivated by efficiency consideration, it lacks the ready interpretability of cluster- or participant-average approaches. Furthermore, it is not an appropriate estimand as it depends on an unknown parameter $\rho^{*}$ , which is specific to particular contexts or datasets. For instance, if we analyse a different outcome (switching from a continuous outcome to a binary outcome), we would expect a different intracluster correlation value $\rho^{*}$ , which would lead to a re-weighting of the participants and clusters in constructing Δ(ρ*) . This would not be the case for the other estimands defined earlier as those weighting schemes are independent of the outcome used. Hence, choosing Δ(ρ*) as an estimand implicitly means that the estimand will also be dictated by the data we analyse rather than the scientific question alone. Under informative cluster size, this estimand generally differs from the estimands we defined in Section 2.4, except for two extreme cases. That is, when ρ*=0 , we have $\Delta (\rho *)=\Delta^{CS-PA}=\Delta^{MG-PA}$ , and when ρ*=1 , we have $\Delta (\rho *)=\Delta^{CS-CA}=\Delta^{MG-CA}$ . However, these two cases are generally unlikely to hold for most CRTs. As such, mixed models may generally be biased for the cluster-specific, participant-average effect when there is an informative cluster size. Of note, they will also be biased for other estimands as well, such as the cluster-specific, cluster-average effect, when there is informative cluster size.

However, in the absence of informative cluster size, linear mixed-effects models can provide a consistent estimator for the ‘difference’ summary measure for all four estimands, as in this case, the values of all estimands, $\Delta (\rho^{*}),\text{\textbackslash{}; }\Delta^{MG-PA},\text{\textbackslash{}; }\Delta^{MG-CA},\text{\textbackslash{}; }\Delta^{CS-PA},\text{\textbackslash{}; }\Delta^{CS-CA}$ , will coincide (strictly speaking, they all coincide under the super-population perspective, whereas their differences vanish with M→∞ under the finite-population perspective). Wang et al.^47^ provide a detailed treatment of the robustness of linear mixed-effects models for CRTs under arbitrary model misspecifications in the absence of informative cluster size.

However, these results do not easily generalise to other link functions such as the logistic mixed-effects models, since the marginal likelihood and the score equations are analytically intractable. Therefore, the requirement for consistent estimation of the OR estimands (defined in Section 2) with logistic mixed-effects models is likely more stringent compared to linear mixed-effects models. To this end, an important area of future research is around whether deviations from model assumptions (e.g. that the normality assumption for the random effects in the logistics mixed-effects model is misspecified) may affect the consistent estimation of all four OR estimands.

### 3.4 GEEs with an exchangeable correlation structure

GEEs are applied to individual participant data.^45^ They involve specifying a working correlation structure in conjunction with cluster-robust SEs. Typically, for CRTs, an exchangeable working correlation structure is specified, that is, the correlation is assumed to be constant between any two participants in the same cluster, and 0 between participants from different clusters. The use of the cluster-robust SE ensures consistent variance estimation, even when the working correlation structure is misspecified. For a difference in means, GEEs take the following mean model:

(18) $$E(Y_{ij})=\alpha +\beta Z_{j}$$

and a working correlation structure is specified for $(Y_{1j},\ldots ,Y_{n_{j},j}{)}^{\prime }$ for each cluster. Estimation is done using a pair of estimating equations, one for the mean parameters and one for the correlation parameters.^45^

For an OR summary measure, the following form is used:

(19) $$\mathrm{logit}(P\{Y_{ij}=1\})=\alpha +\beta Z_{j}$$

GEEs with an exchangeable correlation structure have been conventionally considered as tools to estimate a marginal, participant-average effect. However, in the presence of informative cluster size, just like mixed-effects models, they do not in fact give equal weight to each participant, and as such, they require non-informative cluster size to provide consistent estimation of estimands defined earlier.^19^ See, for example, Wang et al.^19^ for a detailed overview of the robustness of GEEs with an exchangeable correlation structure and an identity link function.

## 4. Application to the randomised evaluation of sedation titration for respiratory failure (RESTORE) trial

The RESTORE trial was a CRT that compared protocolised sedation with usual care in critically ill children who were mechanically ventilated for acute respiratory failure.^48^ Thirty-one clusters were randomised, with the number of participants in each cluster ranging between 12 and 272. In total, 2449 participants were enrolled.

We focus on the adverse event ‘post-extubation stridor’, which denoted the presence of inspiratory noise indicating the narrowing of the airway (yes vs. no). Our aims were (i) to compare estimators for the same estimand, to determine to what extent different choices may impact results; and (ii) to compare estimators across different estimands, to evaluate to what extent choice of estimand may affect interpretation of trial results.

We implemented each of the estimators described in Section 3. For IEEs and GEEs, we calculated cluster-robust SEs using the Fay–Graubard small-sample correction.^49^ For the analysis of cluster-level summaries, we used Huber–White SEs, and for mixed-effects models, we used model-based SEs.

### 4.1 Difference between estimators

**Figure 1.**
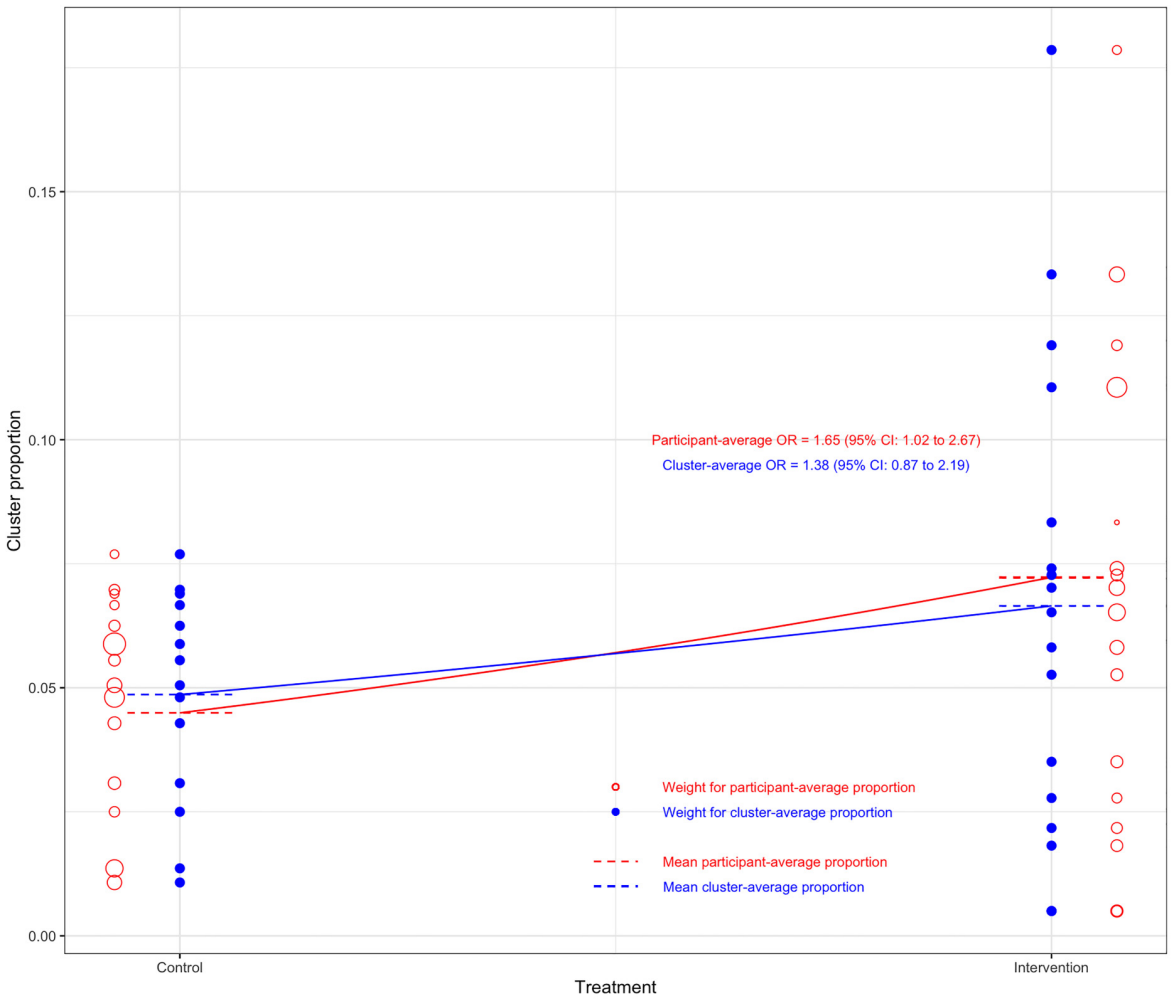
Difference between marginal participant- versus cluster-average odds ratio for ‘post-extubation stridor’ in the randomised evaluation of sedation titration for respiratory failure (RESTORE) trial. Each bubble denotes the proportion of events in that cluster. The size of the bubbles represents the weight given to each cluster, with hollow bubbles representing the participant-average weighting, and solid bubbles denoting the cluster-average weighting. The overall treatment group means are closer together under the cluster-average weighting than the participant-average weighting, owing to the cluster-average weighting giving more weight to smaller clusters with smaller between-group differences.

Results are shown in Table 4 and Figure 1. The estimated OR ranged substantially across different estimands, from 1.38 (95% CI 0.87 to 2.19, *p* = 0.17) for the marginal cluster-average effect, to 1.83 (95% CI 1.06 to 3.14, *p* = 0.03) for the cluster-specific participant-average effect.

**Table 4.** Re-analysis results for the RESTORE trial for the outcome ‘post-extubation stridor’.

Estimand	Estimator	Odds ratio (95% CI)	*P*-value
Marginal, participant-average			
	IEEs (unweighted)^a^	1.65 (1.02 to 2.67)	0.04
	Cluster-level summaries (weighted)^b^	1.65 (1.08 to 2.51)	0.02
	GEEs with exchangeable correlation (unweighted)^a^	1.57 (0.98 to 2.50)	0.06
Cluster-specific, participant-average			
	Cluster-level summaries (weighted)^b^	1.83 (1.06 to 3.14)	0.03
	Mixed-effects logistic regression model	1.54 (0.97 to 2.44)	0.07
Marginal, cluster-average			
	IEEs (weighted)^a^	1.38 (0.87 to 2.19)	0.17
	Cluster-level summaries (unweighted)^b^	1.38 (0.89 to 2.14)	0.15
Cluster-specific, cluster-average			
	Cluster-level summaries (unweighted)^b^	1.51 (0.90 to 2.52)	0.11

RESTORE: randomised evaluation of sedation titration for respiratory failure; 95% CI: 95% confidence interval; GEE: generalised estimating equation; IEE: independence estimating equation.

Odds ratios are for the comparison of intervention versus control. This trial enrolled 31 clusters, and the event rate was 7.2% in the intervention versus 4.5% in the control. Intra-class correlation coefficient = 0.03. *P*-value for interaction between treatment and cluster size ≥100 = 0.21. Marginal, participant-average odds ratio = 1.15 (0.56 to 2.36) in clusters of size <100, and 2.26 (1.49 to 3.43) in clusters of size ≥100.

^a^CIs were calculated using cluster-robust standard errors with the Fay–Graubard correction.

^b^CIs were calculated using Huber–White standard errors.

The choice of both estimand and estimator impacted conclusions. Estimates for the participant-average estimands (both marginal and cluster-specific) were larger than those for the cluster-average estimands and were statistically significant (based on a 0.05 significance level), while those for the cluster-average estimands were not.

However, only specific estimators for the two participant-average estimands demonstrated statistical significance. In particular, mixed-effects models and GEEs with an exchangeable correlation structure produced smaller estimates of treatment effect than IEEs or the analysis of cluster-level summaries, and, as a consequence, results from mixed-effects models and GEEs with an exchangeable correlation structure were not statistically significant, while those from IEEs and cluster-level summaries were. For example, for the marginal participant-average effect, IEEs provided an estimated OR of 1.65 (95% CI 1.02 to 2.67, *p* = 0.04), while GEEs with an exchangeable correlation structure produced an estimated OR of 1.57 (95% CI 0.98 to 2.50, *p* = 0.06). Similarly, for the cluster-specific participant-average effect, the use of weighted cluster-level summaries provided an OR of 1.83 (95% CI 1.06 to 3.14, *p* = 0.03), while a mixed-effects logistic regression model gave an estimated OR of 1.54 (95% CI 0.97 to 2.44, *p* = 0.07).

The participant-average effects were larger than the corresponding cluster-average effects, which is consistent with the implication of informative cluster size.^50^ Smaller clusters had numerically smaller treatment effects than larger clusters: the participant-average OR was 1.15 (95% CI 0.56 to 2.36) in the 24 clusters of size <100, while it was 2.26 (95% CI 1.49 to 3.43) in the seven clusters of size ≥100. We also observed attenuated estimates from mixed-effects models and GEEs with an exchangeable correlation structure. Although often thought to estimate participant-average effects, in fact, these models can give more weight to smaller clusters. Hence, they may give an estimate ‘shifted’ towards the cluster-average effect, in this case, a smaller overall treatment effect.^4^ However, the interaction between small and large clusters was not statistically significant (*p* = 0.21), so we cannot definitively conclude there was informative cluster size in this setting.

## 5. Discussion

The use of estimands to clarify the interpretation of treatment effects and ensure that estimators are aligned with study objectives has rapidly been gaining attention in randomised trials; however, most research has been focused on individually randomised trials. CRTs have unique features that require additional specification in the estimand definition. In this paper, we have: (i) defined estimands that encompass both the marginal versus cluster-specific and participant- versus cluster-average attributes together; and (ii) described commonly used, simple estimators for each of these estimands.

Our re-analysis of the RESTORE trial demonstrated the value of careful consideration of both the estimand and the estimator. Different estimands led to different conclusions around the effect of treatment (e.g. OR = 1.38, *p* = 0.17 for the marginal cluster-average effect vs. OR = 1.83, *p* = 0.03 for the cluster-specific participant-average effect). Similarly, different estimators of the same estimand also affected interpretation. Mixed-effects models and GEEs with an exchangeable correlation structure, which may be considered for estimation of participant-average effects (cluster-specific and marginal, respectively), provided lower estimates of treatment effect that were closer to the cluster-average effect than either IEEs or the analysis of cluster-level summaries. This also led to a change in statistical significance. As such, careful consideration around the plausibility of the assumptions required by each estimator is essential.

The choice of estimand should be driven by the trial objectives. We anticipate all four estimands described in Table 2 may be of interest, depending on the specific study objectives. For instance, if interest lies in understanding how well clusters have implemented the intervention (as measured by adherence), a cluster-specific and/or cluster-average estimand may be more appropriate. Conversely, if interest lies in understanding the absolute number of patients that would be saved by the intervention, a marginal participant-average estimand may be most appropriate.^4^ We acknowledge that the choice of estimand may not always be straightforward, but this should not discourage conversations between the statisticians and investigators around which estimands may be most appropriate in a given CRT. Further work describing when different estimands would be most appropriate, and using case studies to describe how investigators chose their estimand, would be of value.^4^

In this paper, we have defined four estimands that could be used in CRTs. However, the estimands described here are not exhaustive; alternative estimands could be defined, for instance, by using different weighting schemes than those proposed here. While we feel that the participant- and cluster-average estimands (which give equal weight to participants or clusters) lead to clinically interpretable treatment effects that align well with standard estimators used in CRTs, we acknowledge that other approaches may be of interest to investigators. It is not our intention that investigators must use one of the estimands defined within this paper; in our view, the most important thing is to have a well-defined estimand that is clinically justified based on the study's objectives, and, importantly, this paper provides a coherent framework with standard terminology to enable investigators to describe their estimand, regardless of whether it is one of those described here.

This article suggests a number of areas of future research. For example, we have focused on leveraging the asymptotic properties of each estimator. However, many CRTs only enrol a small number of clusters.^23^ Thus, evaluation of the properties of these estimators for well-defined estimands in small-sample settings would be useful, for instance, by extending previous simulation studies to evaluate these estimators under settings that include informative cluster size^15,18,24,51^). In particular, it may be useful to undertake a comprehensive comparison of the benefits and drawbacks of estimators that require an assumption of ‘non-informative cluster size’ (i.e. mixed-effects models and GEEs with an exchangeable correlation structure) versus those that do not (i.e. IEEs and cluster-level summaries). In addition, we have primarily focused on simple parallel-arm CRTs, but longitudinal CRTs with multiple periods are increasingly common. The extension of the estimands in this article to more complex designs, such as the stepped wedge designs,^52,53^ may be of interest.

It would also be useful to evaluate the performance of individual methods. For example, evaluation of different variance estimators along with small-sample corrections for the cluster-level analysis approaches would be useful given the model-based variance functions may be misspecified. Additionally, the marginal cluster-level summary estimator we have described has not been extensively studied, and empirical evaluation using simulations (with and without baseline covariates) would be useful.

## 6. Conclusions

Estimands can help clarify research objectives and ensure appropriate statistical estimators are chosen. In CRTs, additional attributes of the estimand must be specified compared to individually randomised trials (including whether treatment effects are marginal or cluster-specific, and whether they are participant-average or cluster-average). The choice of these attributes should be based on clinical considerations, and an estimator targeted to the chosen estimand should be used to ensure estimand-aligned statistical analysis of CRTs.

## Appendix

For completeness, we sketch the proof for consistency of each estimator based on the cluster-level summary statistics in Section 3.2. Of note, the consistency of the IEE estimators in Section 3.1 is provided in Wang et al.^19^ (for difference in means) and Zhu et al.^43^ (for OR), and therefore we omit them for brevity. Furthermore, the proof we provide below is based on the super-population perspective as the steps are simpler, relatively more standard in the CRTs literature, and are more intuitive to understand. The proof under the finite-population perspective requires taking an expectation over the randomisation distribution and invoking the less familiar finite-population, design-based results.^41^ Therefore, we only focus on the super-population proof. The consistent results, however, do not change regardless of which of these two perspectives is used.

### A.1 Consistency of the marginal, participant-average estimator

Recall that we consider a cluster size weighted GLM for the cluster-specific summary statistic ${\hat{\pi }}_{j}$ as $\text{logit}({\hat{\pi }}_{j})=\alpha +\beta Z_{j}$ . Below we consider an arbitrary family specification which corresponds to an arbitrary variance function $\nu (Z_{j})$ . This variance function is at most a function of treatment; for example, $\nu (Z_{j})\propto 1$ if the family is specified as Gaussian. Under these conditions, the 2×1 estimating equations can be written out in explicit forms as:

$$\overset{M}{\underset{j=1}{\sum }}\;\left(\begin{array}{c} 1\\ Z_{j} \end{array}\right)\nu (Z_{j})\left(\frac{\exp\;(\alpha +\beta Z_{j})}{{\left\{1+\exp\;(\alpha +\beta Z_{j})\right\}}^{2}}\right)\left(n_{j}{\hat{\pi }}_{j}-n_{j}\frac{\exp\;(\alpha +\beta Z_{j})}{1+\exp\;(\alpha +\beta Z_{j})}\right)=0$$

The first row of this equation is

$$\begin{array}{rl} 0 & =\overset{M}{\underset{j=1}{\sum }}\;\nu (Z_{j})\left(\frac{\exp\;(\alpha +\beta Z_{j})}{\{{1+\exp\;(\alpha +\beta Z_{j})\}}^{2}}\right)\left(n_{j}{\hat{\pi }}_{j}-n_{j}\frac{\exp\;(\alpha +\beta Z_{j})}{1+\exp\;(\alpha +\beta Z_{j})}\right)\\ & =\overset{M}{\underset{j=1}{\sum }}\;Z_{j}\nu (1)\left(\frac{\exp\;(\alpha +\beta )}{{\left\{1+\exp\;(\alpha +\beta )\right\}}^{2}}\right)\left(n_{j}{\hat{\pi }}_{j}-n_{j}\frac{\exp\;(\alpha +\beta )}{1+\exp\;(\alpha +\beta )}\right)\\ & \;+\overset{M}{\underset{j=1}{\sum }}\;(1-Z_{j})\nu (0)\left(\frac{\exp\;(\alpha )}{\{1+\exp\;(\alpha ){\}}^{2}}\right)\left(n_{j}{\hat{\pi }}_{j}-n_{j}\frac{\exp\;(\alpha )}{1+\exp\;(\alpha )}\right) \end{array}$$

Combining with the second row, and since ν(1) and ν(0) are both constant regardless of the choice of family, they can be omitted and we have:

$$\begin{array}{r} \overset{M}{\underset{j=1}{\sum }}\;Z_{j}\nu (1)\left(\frac{\exp\;(\alpha +\beta )}{{\left\{1+\exp\;(\alpha +\beta )\right\}}^{2}}\right)\left(n_{j}{\hat{\pi }}_{j}-n_{j}\frac{\exp\;(\alpha +\beta )}{1+\exp\;(\alpha +\beta )}\right)=\overset{M}{\underset{j=1}{\sum }}\;Z_{j}\left(n_{j}{\hat{\pi }}_{j}-n_{j}\frac{\exp\;(\alpha +\beta )}{1+\exp\;(\alpha +\beta )}\right)=0\\ \overset{M}{\underset{j=1}{\sum }}\;(1-Z_{j})\nu (0)\left(\frac{\exp\;(\alpha )}{{\left\{1+\exp\;(\alpha )\right\}}^{2}}\right)\left(n_{j}{\hat{\pi }}_{j}-n_{j}\frac{\exp\;(\alpha )}{1+\exp\;(\alpha )}\right)=\overset{M}{\underset{j=1}{\sum }}\;(1-Z_{j})\left(n_{j}{\hat{\pi }}_{j}-n_{j}\frac{\exp\;(\alpha )}{1+\exp\;(\alpha )}\right)=0 \end{array}$$

Solving these two equations for the parameters and rearranging terms, we obtain the form of the estimator as:

$$\hat{\beta }=\text{logit}\left\{\frac{\sum_{j=1}^{M}\;Z_{j}n_{j}{\hat{\pi }}_{j}}{\sum_{j=1}^{M}\;Z_{j}n_{j}}\right\}-\text{logit}\left\{\frac{\sum_{j=1}^{M}\;(1-Z_{j})n_{j}{\hat{\pi }}_{j}}{\sum_{j=1}^{M}\;(1-Z_{j})n_{j}}\right\}=\text{logit}\{{\hat{P}}_{1}\}-\text{logit}\{{\hat{P}}_{0}\}$$

Now observe that, as the number of clusters increases to infinity ( M→∞ )

$${\hat{P}}_{1}=\frac{M^{-1}\sum_{j=1}^{M}\;\sum_{i=1}^{n_{j}}\;Z_{j}Y_{ij}}{M^{-1}\sum_{j=1}^{M}\;Z_{j}n_{j}}\overset{p}{\to }\;\frac{E\left[\sum_{i=1}^{n_{j}}\;Z_{j}Y_{ij}\right]}{E[Z_{j}n_{j}]}=\frac{E(Z_{j})E\left[\sum_{i=1}^{n_{j}}\;Y_{ij}^{\left(1\right)}\right]}{E[Z_{j}]E(n_{j})}=\frac{E\left[\sum_{i=1}^{n_{j}}\;Y_{ij}^{\left(1\right)}\right]}{E[n_{j}]}$$

where the convergence in probability statement results from an application of the Weak Law of Large Numbers for independent but non-identically distributed data and the Continuous Mapping Theorem. The subsequent equality is due to cluster-level randomisation. Following the exact same steps under control, we observe that:

$${\hat{P}}_{0}\overset{p}{\to }\;\frac{E\left[\sum_{i=1}^{n_{j}}\;Y_{ij}^{\left(0\right)}\right]}{E[n_{j}]}$$

and therefore, by the Continuous Mapping Theorem:

$$\exp\;(\hat{\beta })\overset{p}{\to }\;\frac{E\left(\sum_{i=1}^{n_{j}}\;Y_{ij}^{\left(1\right)}\right)/E(n_{j})/\left(1-E\left(\sum_{i=1}^{n_{j}}\;Y_{ij}^{\left(1\right)}\right)/E(n_{j})\right)}{E\left(\sum_{i=1}^{n_{j}}\;Y_{ij}^{\left(0\right)}\right)/E(n_{j})/\left(1-E\left(\sum_{i=1}^{n_{j}}\;Y_{ij}^{\left(0\right)}\right)/E(n_{j})\right)}=\Gamma^{MG-PA}$$

Clearly, since $v(Z_{j})$ does not enter into the estimating equations and hence the treatment effect estimator, the choice of family has no impact on the final treatment effect estimator.

### A.2 Consistency of the marginal, cluster-average estimator

To find the form of the estimator of the unweighted cluster-level GLM, we simply remove the cluster size weight in the estimating equation of Section A.1, and obtain:

$$\overset{M}{\underset{j=1}{\sum }}\;\left(\begin{array}{c} 1\\ Z_{j} \end{array}\right)\nu (Z_{j})\left(\frac{\exp\;(\alpha +\beta Z_{j})}{\{1+\exp\;(\alpha +\beta Z_{j}){\}}^{2}}\right)\left({\hat{\pi }}_{j}-\frac{\exp\;(\alpha +\beta Z_{j})}{1+\exp\;(\alpha +\beta Z_{j})}\right)=0$$

Since $v(Z_{j})$ is only at most a function of the treatment indicator, the above equation then implies:

$$\begin{array}{rl} \sum_{j=1}^{M}Z_{j}\left({\hat{\pi }}_{j}-\frac{\exp\;(\alpha +\beta )}{1+\exp\;(\alpha +\beta )}\right) & =0 \end{array}$$

$$\begin{array}{rl} \sum_{j=1}^{M}(1-Z_{j})\left({\hat{\pi }}_{j}-\frac{\exp\;(\alpha )}{1+\exp\;(\alpha )}\right) & =0 \end{array}$$

and the treatment effect estimator is given explicitly as:

$$\begin{array}{rl} \hat{\beta } & =\text{logit}\left\{\frac{\sum_{j=1}^{M}\;Z_{j}{\hat{\pi }}_{j}}{\sum_{j=1}^{M}\;Z_{j}}\right\}-\text{logit}\left\{\frac{\sum_{j=1}^{M}\;(1-Z_{j}){\hat{\pi }}_{j}}{\sum_{j=1}^{M}\;(1-Z_{j})}\right\}\\ & =\text{logit}{\{\hat{P}}_{1}\}-\text{logit}\{{\hat{P}}_{0}\}\overset{p}{\to }\;\text{logit}\left\{E\left(\frac{\sum_{i=1}^{n_{j}}\;Y_{ij}^{\left(1\right)}}{n_{j}}\right)\right\}\\ & \;-\text{logit}\left\{E\left(\frac{\sum_{i=1}^{n_{j}}\;Y_{ij}^{\left(0\right)}}{n_{j}}\right)\right\} \end{array}$$

where the convergence in probability statements assumes the number of clusters M→∞ , and is a result of the appropriate Weak Law of Large Numbers and the Continuous Mapping Theorem. Therefore:

$$\exp\;(\hat{\beta })\overset{p}{\to }\;\frac{E\left(\sum_{i=1}^{n_{j}}\;Y_{ij}^{\left(1\right)}/n_{j}\right)/\left(1-E\left(\sum_{i=1}^{n_{j}}\;Y_{ij}^{\left(1\right)}/n_{j}\right)\right)}{E\left(\sum_{i=1}^{n_{j}}\;Y_{ij}^{\left(0\right)}/n_{j}\right)/\left(1-E\left(\sum_{i=1}^{n_{j}}\;Y_{ij}^{\left(0\right)}/n_{j}\right)\right)}=\Gamma^{MG-CA}$$

Similarly, the choice of family has no impact on the final treatment effect estimator.

### A.3 Consistency of the cluster-specific, participant-average estimator

Recall that a cluster size weighted linear regression is fitted to the log OR summary in each cluster, such that a working model is given by $\log\;[{\hat{\pi }}_{j}/(1-{\hat{\pi }}_{j})]=\alpha +\beta Z_{j}+\varepsilon_{j}$ in Section 3.2.3. Denote the M×2 design matrix as $D=(1_{M},Z)$ , where $1_{M}$ is an M -vector and $Z=(Z_{1},\ldots ,Z_{M}{)}^{\prime }$ is the vector of treatment indicators; define ${\hat{Y}}^{smr}=({\hat{Y}}_{1}^{smr},\ldots ,{\hat{Y}}_{M}^{smr}{)}^{\prime }={\log\;[{\hat{\pi }}_{1}/(1-{\hat{\pi }}_{1})],\ldots ,\log\;[{\hat{\pi }}_{M}/(1-{\hat{\pi }}_{M})]}^{\prime }$ as the vector of responses, and $W=\text{diag}(n_{j})$ as the weight matrix. Then we have:

$$(D^{\prime }WD{)}^{-1}={\left(\begin{array}{cc} N & \sum_{j=1}^{M}\;Z_{j}n_{j}\\ \sum_{j=1}^{M}\;Z_{j}n_{j} & \sum_{j=1}^{M}\;Z_{j}n_{j} \end{array}\right)}^{-1}=\frac{1}{N\sum_{j=1}^{M}\;Z_{j}n_{j}-{\left(\sum_{j=1}^{M}\;Z_{j}n_{j}\right)}^{2}}\left(\begin{array}{cc} \sum_{j=1}^{M}\;Z_{j}n_{j} & -\sum_{j=1}^{M}\;Z_{j}n_{j}\\ -\sum_{j=1}^{M}\;Z_{j}n_{j} & N \end{array}\right)$$

$$D^{\prime }W{\hat{Y}}^{smr}=\left(\begin{array}{c} \sum_{j=1}^{M}\;{\hat{Y}}_{j}^{smr}n_{j}\\ \sum_{j=1}^{M}\;Z_{j}{\hat{Y}}_{j}^{smr}n_{j} \end{array}\right)$$

Therefore, the treatment coefficient estimator $\hat{\beta }$ is the last element of the weighted least squares formula, given by:

(20)
$$\begin{array}{rl} \hat{\beta } & =\{{\left(D^{\prime }WD\right)}^{-1}(D^{\prime }W{\hat{Y}}^{smr}){\}}_{\left[2,1\right]}=\frac{N\sum_{j=1}^{M}\;Z_{j}{\hat{Y}}_{j}^{smr}n_{j}-\left(\sum_{j=1}^{M}\;{\hat{Y}}_{j}^{smr}n_{j}\right)\left(\sum_{j=1}^{M}\;Z_{j}n_{j}\right)}{N\sum_{j=1}^{M}\;Z_{j}n_{j}-{\left(\sum_{j=1}^{M}\;Z_{j}n_{j}\right)}^{2}}\\ & =\frac{\left(\sum_{j=1}^{M}\;(1-Z_{j})n_{j}\right)\left(\sum_{j=1}^{M}\;Z_{j}{\hat{Y}}_{j}^{smr}n_{j}\right)-\left(\sum_{j=1}^{M}\;(1-Z_{j}){\hat{Y}}_{j}^{smr}n_{j}\right)\left(\sum_{j=1}^{M}\;Z_{j}n_{j}\right)}{\left(\sum_{j=1}^{M}\;Z_{j}n_{j}\right)\left(\sum_{j=1}^{M}\;(1-Z_{j})n_{j}\right)}\\ & =\frac{\sum_{j=1}^{M}\;Z_{j}{\hat{Y}}_{j}^{smr}n_{j}}{\sum_{j=1}^{M}\;Z_{j}n_{j}}-\frac{\sum_{j=1}^{M}\;(1-Z_{j}){\hat{Y}}_{j}^{smr}n_{j}}{\sum_{j=1}^{M}\;(1-Z_{j})n_{j}} \end{array}$$

Define:

$$\text{ODD}{\text{S}}_{j}(z)=\frac{1}{n_{j}}\overset{n_{j}}{\underset{i=1}{\sum }}\;Y_{ij}^{\left(z\right)}/\left(1-\frac{1}{n_{j}}\overset{n_{j}}{\underset{i=1}{\sum }}\;Y_{ij}^{\left(z\right)}\right)$$

and therefore $\text{O}{\text{R}}_{j}=\text{ODD}{\text{S}}_{j}(1)/\text{ODD}{\text{S}}_{j}(0)$ . Under the consistency assumption,

${\hat{Y}}_{j}^{smr}=\log\;\left(\frac{{\hat{\pi }}_{j}}{1-{\hat{\pi }}_{j}}\right)=Z_{j}\log\;(\text{ODD}{\text{S}}_{j}(1))+(1-Z_{j})\log\;(\text{ODD}{\text{S}}_{j}(0))$ , and therefore (20) becomes

$$\begin{array}{rl} \hat{\beta } & =\frac{\sum_{j=1}^{M}\;Z_{j}\log\;(\text{ODD}{\text{S}}_{j}(1))n_{j}}{\sum_{j=1}^{M}\;Z_{j}n_{j}}-\frac{\sum_{j=1}^{M}\;(1-Z_{j})\log\;(\text{ODD}{\text{S}}_{j}(0))n_{j}}{\sum_{j=1}^{M}\;(1-Z_{j})n_{j}}\\ & \;\overset{p}{\to }\;\frac{E[Z_{j}\log\;(\text{ODD}{\text{S}}_{j}(1))n_{j}]}{E[Z_{j}n_{j}]}-\frac{E[(1-Z_{j})\log\;(\text{ODD}{\text{S}}_{j}(0))n_{j}]}{E[(1-Z_{j})n_{j}]}\\ & =\frac{E[n_{j}\left\{\log\;(\text{ODD}{\text{S}}_{j}(1)/\text{ODD}{\text{S}}_{j}(0))\right\}]}{E[n_{j}]}=\frac{E[n_{j}\{\log\;(\text{O}{\text{R}}_{j})\}]}{E[n_{j}]}=\log\;(\Gamma^{CS-PA}) \end{array}$$

In the above, the convergence in the probability statement results from an application of the Weak Law of Large Numbers for independent but non-identically distributed data, the subsequent equality is due to the randomisation of $Z_{j}$ such that $EZ_{j}\log\;[\text{ODD}{\text{S}}_{j}(1)]n_{j}=E(Z_{j})E\log\;[\text{ODD}{\text{S}}_{j}(1)]n_{j}$ and $E[Z_{j}n_{j}]=E(Z_{j})E[n_{j}]$ , etc. Then by the Continuous Mapping Theorem, we have $\exp\;(\hat{\beta })\overset{p}{\to }\;\Gamma^{CS-PA}$ .

### A.4 Consistency of the cluster-specific, cluster-average estimator

The proof of consistency follows from Section A.3 by replacing $n_{j}=1$ for all *j*. Therefore, we obtain that:

$$\hat{\beta }=\frac{\sum_{j=1}^{M}\;Z_{j}\log\;(\text{ODD}{\text{S}}_{j}(1))}{\sum_{j=1}^{M}\;Z_{j}}-\frac{\sum_{j=1}^{M}\;(1-Z_{j})\log\;(\text{ODD}{\text{S}}_{j}(0))}{\sum_{j=1}^{M}\;(1-Z_{j})}\overset{p}{\to }\;E[\log\;(\text{O}{\text{R}}_{j})]=\log\;(\Gamma^{CS-CA})$$

This then leads to $\exp\;(\hat{\beta })\overset{p}{\to }\;\Gamma^{CS-CA}$ under the Continuous Mapping Theorem.
