# Supplementary material for: Demystifying estimands in cluster-randomised trials
Source: Stat Methods Med Res. Author manuscript; Available in PMC 2024 Aug 28. (PMC11348634; doi:10.1177/09622802241254197)
Supplement: Appendix [file EMS197737-supplement-Appendix.pdf]

Appendix

For completeness, we sketch the proof for consistency of each estimator based on the cluster-level summary statistics in Section 3.2. Of note, the consistency of the IEE estimators in Section 3.1 is provided in Wang et al.[19] (for difference in means) and Zhu et al.[43] (for OR), and therefore we omit them for brevity. Furthermore, the proof we provide below is based on the super-population perspective as the steps are simpler, relatively more standard in the CRTs literature, and

are more intuitive to understand. The proof under the finite-population perspective requires taking an expectation over the randomisation distribution and invoking the less familiar finite-population, design-based results.[41] Therefore, we only focus on the super-population proof. The consistent results, however, do not change regardless of which of these two perspectives is used.

A.1 Consistency of the marginal, participant-average estimator

Recall that we consider a cluster size weighted GLM for the cluster-specific summary statistic $\hat{\pi}_j$ as $\text{logit}(\hat{\pi}_j) = \alpha + \beta Z_j$. Below we consider an arbitrary family specification which corresponds to an arbitrary variance function $v(Z_j)$. This variance function is at most a function of treatment; for example, $v(Z_j) \propto 1$ if the family is specified as Gaussian. Under these conditions, the 2×1 estimating equations can be written out in explicit forms as:

$$\sum_{j=1}^{M} \begin{pmatrix} 1 \\ Z_j \end{pmatrix} v(Z_j) \left(\frac{\exp(\alpha + \beta Z_j)}{\{1 + \exp(\alpha + \beta Z_j)\}^2} \right) \left(n_j \hat{\pi}_j - n_j \frac{\exp(\alpha + \beta Z_j)}{1 + \exp(\alpha + \beta Z_j)} \right) = 0$$

The first row of this equation is

$$\begin{aligned} 0 &= \sum_{j=1}^{M} v(Z_j) \left(\frac{\exp(\alpha + \beta Z_j)}{\{1 + \exp(\alpha + \beta Z_j)\}^2} \right) \left(n_j \hat{\pi}_j - n_j \frac{\exp(\alpha + \beta Z_j)}{1 + \exp(\alpha + \beta Z_j)} \right) \\ &= \sum_{j=1}^{M} Z_j v(1) \left(\frac{\exp(\alpha + \beta)}{\{1 + \exp(\alpha + \beta)\}^2} \right) \left(n_j \hat{\pi}_j - n_j \frac{\exp(\alpha + \beta)}{1 + \exp(\alpha + \beta)} \right) \\ &\quad + \sum_{j=1}^{M} (1 - Z_j) v(0) \left(\frac{\exp(\alpha)}{\{1 + \exp(\alpha)\}^2} \right) \left(n_j \hat{\pi}_j - n_j \frac{\exp(\alpha)}{1 + \exp(\alpha)} \right) \end{aligned}$$

Combining with the second row, and since $v(1)$ and $v(0)$ are both constant regardless of the choice of family, they can be omitted and we have:

$$\sum_{j=1}^{M} Z_j v(1) \left(\frac{\exp(\alpha + \beta)}{\{1 + \exp(\alpha + \beta)\}^2} \right) \left(n_j \hat{\pi}_j - n_j \frac{\exp(\alpha + \beta)}{1 + \exp(\alpha + \beta)} \right) = \sum_{j=1}^{M} Z_j \left(n_j \hat{\pi}_j - n_j \frac{\exp(\alpha + \beta)}{1 + \exp(\alpha + \beta)} \right) = 0$$

$$\sum_{j=1}^{M} (1 - Z_j) v(0) \left(\frac{\exp(\alpha)}{\{1 + \exp(\alpha)\}^2} \right) \left(n_j \hat{\pi}_j - n_j \frac{\exp(\alpha)}{1 + \exp(\alpha)} \right) = \sum_{j=1}^{M} (1 - Z_j) \left(n_j \hat{\pi}_j - n_j \frac{\exp(\alpha)}{1 + \exp(\alpha)} \right) = 0$$

Solving these two equations for the parameters and rearranging terms, we obtain the form of the estimator as:

$$\hat{\beta} = \text{logit} \left\{ \frac{\sum_{j=1}^{M} Z_j n_j \hat{\pi}_j}{\sum_{j=1}^{M} Z_j n_j} \right\} - \text{logit} \left\{ \frac{\sum_{j=1}^{M} (1 - Z_j) n_j \hat{\pi}_j}{\sum_{j=1}^{M} (1 - Z_j) n_j} \right\} = \text{logit}\{\hat{P}_1\} - \text{logit}\{\hat{P}_0\}$$

Now observe that, as the number of clusters increases to infinity ($M \to \infty$)

$$\hat{P}_1 = \frac{M^{-1} \sum_{j=1}^{M} \sum_{i=1}^{n_j} Z_j Y_{ij}}{M^{-1} \sum_{j=1}^{M} Z_j n_j} \xrightarrow{p} \frac{E\left[\sum_{i=1}^{n_j} Z_j Y_{ij}\right]}{E[Z_j n_j]} = \frac{E(Z_j) E\left[\sum_{i=1}^{n_j} Y_{ij}^{(1)}\right]}{E[Z_j] E(n_j)} = \frac{E\left[\sum_{i=1}^{n_j} Y_{ij}^{(1)}\right]}{E[n_j]}$$

where the convergence in probability statement results from an application of the Weak Law of Large Numbers for independent but non-identically distributed data and the Continuous Mapping Theorem. The subsequent equality is due to cluster-level randomisation. Following the exact same steps under control, we observe that:

$$\hat{P}_0 \xrightarrow{p} \frac{E\left[\sum_{i=1}^{n_j} Y_{ij}^{(0)}\right]}{E[n_j]}$$

and therefore, by the Continuous Mapping Theorem:

$$\exp(\hat{\beta}) \xrightarrow{p} \frac{E\left(\sum_{i=1}^{n_j} Y_{ij}^{(1)}\right)/E(n_j)/\left(1 - E\left(\sum_{i=1}^{n_j} Y_{ij}^{(1)}\right)/E(n_j)\right)}{E\left(\sum_{i=1}^{n_j} Y_{ij}^{(0)}\right)/E(n_j)/\left(1 - E\left(\sum_{i=1}^{n_j} Y_{ij}^{(0)}\right)/E(n_j)\right)} = \Gamma^{MG-PA}$$

Clearly, since $v(Z_j)$ does not enter into the estimating equations and hence the treatment effect estimator, the choice of family has no impact on the final treatment effect estimator.

A.2 Consistency of the marginal, cluster-average estimator

To find the form of the estimator of the unweighted cluster-level GLM, we simply remove the cluster size weight in the estimating equation of Section A.1, and obtain:

$$\sum_{j=1}^{M} \binom{1}{Z_j} v(Z_j) \left(\frac{\exp(\alpha + \beta Z_j)}{\{1 + \exp(\alpha + \beta Z_j)\}^2} \right) \left(\hat{\pi}_j - \frac{\exp(\alpha + \beta Z_j)}{1 + \exp(\alpha + \beta Z_j)} \right) = 0$$

Since $v(Z_j)$ is only at most a function of the treatment indicator, the above equation then implies:

$$\sum_{j=1}^{M} Z_j \left(\hat{\pi}_j - \frac{\exp(\alpha + \beta)}{1 + \exp(\alpha + \beta)} \right) = 0$$
$$\sum_{j=1}^{M} (1 - Z_j) \left(\hat{\pi}_j - \frac{\exp(\alpha)}{1 + \exp(\alpha)} \right) = 0$$

and the treatment effect estimator is given explicitly as:

$$\begin{aligned} \hat{\beta} &= \text{logit}\left\{ \frac{\sum_{j=1}^{M} Z_j \hat{\pi}_j}{\sum_{j=1}^{M} Z_j} \right\} - \text{logit}\left\{ \frac{\sum_{j=1}^{M} (1 - Z_j)\hat{\pi}_j}{\sum_{j=1}^{M} (1 - Z_j)} \right\} \\ &= \text{logit}\{\hat{P}_1\} - \text{logit}\{\hat{P}_0\} \xrightarrow{p} \text{logit}\left\{ E\left(\frac{\sum_{i=1}^{n_j} Y_{ij}^{(1)}}{n_j} \right) \right\} \\ &\quad - \text{logit}\left\{ E\left(\frac{\sum_{i=1}^{n_j} Y_{ij}^{(0)}}{n_j} \right) \right\} \end{aligned}$$

where the convergence in probability statements assumes the number of clusters $M \to \infty$, and is a result of the appropriate Weak Law of Large Numbers and the Continuous Mapping Theorem. Therefore:

$$\exp(\hat{\beta}) \xrightarrow{p} \frac{E\left(\sum_{i=1}^{n_j} Y_{ij}^{(1)}/n_j\right) / \left(1 - E\left(\sum_{i=1}^{n_j} Y_{ij}^{(1)}/n_j\right)\right)}{E\left(\sum_{i=1}^{n_j} Y_{ij}^{(0)}/n_j\right) / \left(1 - E\left(\sum_{i=1}^{n_j} Y_{ij}^{(0)}/n_j\right)\right)} = \Gamma^{MG-CA}$$

Similarly, the choice of family has no impact on the final treatment effect estimator.

A.3 Consistency of the cluster-specific, participant-average estimator

Recall that a cluster size weighted linear regression is fitted to the log OR summary in each cluster, such that a working model is given by $\log[\hat{\pi}_j/(1 - \hat{\pi}_j)] = \alpha + \beta Z_j + \epsilon_j$ in Section 3.2.3. Denote the $M \times 2$ design matrix as $D = (1_M, Z)$, where 1_M is an M-vector and $Z = (Z_1, \ldots, Z_M)'$ is the vector of treatment indicators; define $\hat{Y}^{smr} = (\hat{Y}_1^{smr}, \ldots, \hat{Y}_M^{smr})' =$

$\log[\hat{\pi}_1/(1-\hat{\pi}_1)], \ldots, \log[\hat{\pi}_M/(1-\hat{\pi}_M)]'$ as the vector of responses, and $W = \mathrm{diag}(n_j)$ as the weight matrix. Then we have:

$$(D'WD)^{-1} = \begin{pmatrix} N & \sum_{j=1}^M Z_j n_j \\ \sum_{j=1}^M Z_j n_j & \sum_{j=1}^M Z_j n_j \end{pmatrix}^{-1} = \frac{1}{N\sum_{j=1}^M Z_j n_j - \left(\sum_{j=1}^M Z_j n_j\right)^2} \begin{pmatrix} \sum_{j=1}^M Z_j n_j & -\sum_{j=1}^M Z_j n_j \\ -\sum_{j=1}^M Z_j n_j & N \end{pmatrix}$$

$$D'W\hat{Y}^{smr} = \begin{pmatrix} \sum_{j=1}^M \hat{Y}_j^{smr} n_j \\ \sum_{j=1}^M Z_j \hat{Y}_j^{smr} n_j \end{pmatrix}$$

Therefore, the treatment coefficient estimator $\hat{\beta}$ is the last element of the weighted least squares formula, given by:

$$\begin{aligned}
\hat{\beta} = \{(D'WD)^{-1}(D'W\hat{Y}^{smr})\}_{[2,1]} &= \frac{N\sum_{j=1}^M Z_j \hat{Y}_j^{smr} n_j - \left(\sum_{j=1}^M \hat{Y}_j^{smr} n_j\right)\left(\sum_{j=1}^M Z_j n_j\right)}{N\sum_{j=1}^M Z_j n_j - \left(\sum_{j=1}^M Z_j n_j\right)^2} \\
&= \frac{\left(\sum_{j=1}^M (1-Z_j) n_j\right)\left(\sum_{j=1}^M Z_j \hat{Y}_j^{smr} n_j\right) - \left(\sum_{j=1}^M (1-Z_j)\hat{Y}_j^{smr} n_j\right)\left(\sum_{j=1}^M Z_j n_j\right)}{\left(\sum_{j=1}^M Z_j n_j\right)\left(\sum_{j=1}^M (1-Z_j) n_j\right)} \\
&= \frac{\sum_{j=1}^M Z_j \hat{Y}_j^{smr} n_j}{\sum_{j=1}^M Z_j n_j} - \frac{\sum_{j=1}^M (1-Z_j)\hat{Y}_j^{smr} n_j}{\sum_{j=1}^M (1-Z_j) n_j}
\end{aligned} \tag{20}$$

Define:

$$\mathrm{ODDS}_j(z) = \frac{1}{n_j}\sum_{i=1}^{n_j} Y_{ij}^{(z)} \Big/ \left(1 - \frac{1}{n_j}\sum_{i=1}^{n_j} Y_{ij}^{(z)}\right)$$

and therefore $\mathrm{OR}_j = \mathrm{ODDS}_j(1)/\mathrm{ODDS}_j(0)$. Under the consistency assumption, $\hat{Y}_j^{smr} = \log\left(\frac{\hat{\pi}_j}{1-\hat{\pi}_j}\right) = Z_j \log(\mathrm{ODDS}_j(1)) + (1-Z_j)\log(\mathrm{ODDS}_j(0))$, and therefore (20) becomes

$$\begin{aligned}
\hat{\beta} &= \frac{\sum_{j=1}^M Z_j \log(\mathrm{ODDS}_j(1)) n_j}{\sum_{j=1}^M Z_j n_j} - \frac{\sum_{j=1}^M (1-Z_j)\log(\mathrm{ODDS}_j(0)) n_j}{\sum_{j=1}^M (1-Z_j) n_j} \\
&\xrightarrow{p} \frac{E[Z_j \log(\mathrm{ODDS}_j(1)) n_j]}{E[Z_j n_j]} - \frac{E[(1-Z_j)\log(\mathrm{ODDS}_j(0)) n_j]}{E[(1-Z_j) n_j]} \\
&= \frac{E[n_j\{\log(\mathrm{ODDS}_j(1)/\mathrm{ODDS}_j(0))\}]}{E[n_j]} = \frac{E[n_j\{\log(\mathrm{OR}_j)\}]}{E[n_j]} = \log(\Gamma^{CS-PA})
\end{aligned}$$

In the above, the convergence in the probability statement results from an application of the Weak Law of Large Numbers for independent but non-identically distributed data, the subsequent equality is due to the randomisation of Z_j such that $EZ_j \log[\mathrm{ODDS}_j(1)]n_j = E(Z_j)E\log[\mathrm{ODDS}_j(1)]n_j$ and $E[Z_j n_j] = E(Z_j)E[n_j]$, etc. Then by the Continuous Mapping Theorem, we have $\exp(\hat{\beta}) \xrightarrow{p} \Gamma^{CS-PA}$.

A.4 Consistency of the cluster-specific, cluster-average estimator

The proof of consistency follows from Section A.3 by replacing $n_j = 1$ for all j. Therefore, we obtain that:

$$\hat{\beta} = \frac{\sum_{j=1}^M Z_j \log(\mathrm{ODDS}_j(1))}{\sum_{j=1}^M Z_j} - \frac{\sum_{j=1}^M (1-Z_j)\log(\mathrm{ODDS}_j(0))}{\sum_{j=1}^M (1-Z_j)} \xrightarrow{p} E[\log(\mathrm{OR}_j)] = \log(\Gamma^{CS-CA})$$

This then leads to $\exp(\hat{\beta}) \xrightarrow{p} \Gamma^{CS-CA}$ under the Continuous Mapping Theorem.
